# Synthesis and biological evaluation of novel (*E*)-*N'*-benzylidene hydrazides as novel c-Met inhibitors through fragment based virtual screening

**DOI:** 10.1080/14756366.2019.1702655

**Published:** 2020-01-06

**Authors:** Jing-wei Liang, Shi-long Li, Shan Wang, Wan-qiu Li, Fan-hao Meng

**Affiliations:** School of Pharmacy, China Medical University, Shen Yang, China

**Keywords:** Virtual screening, c-Met inhibitor, QSAR, benzylidene hydrazides

## Abstract

C-Met plays a crucial role in the development and progression of neoplastic disease. Type II c-Met inhibitors recognise the inactive DFG-out conformation of the kinase, result in better anti-tumour effects due to synergistic effect against the other kinases. According to our previous works, an *(E)-N'-*benzylidene group was selected as the initial fragment. Two series of (*E*)-*N'*-benzylidene hydrazides were designed by fragment growth method. The inhibitory activities were *in vitro* investigated against c-Met and VEGFR-2. Compound **10b** exhibited the most potent inhibitory activity against the c-Met inhibitor (IC_50_ = 0.37 nM). Compound **11b** exhibited multi-target c-Met kinase inhibitory activity as a potential type II c-Met inhibitor (IC_50_ = 3.41 nM against c-Met; 25.34 nM against VEGFR-2). The two compounds also demonstrate the feasibility of fragment-based virtual screening method for drug discovery.

## 1. Introduction

Hepatocyte growth factor receptor (HGFR/c-Met) is a transmembrane heterodimer comprising two disulphide-linked chains, including an outer α-chain (50 kDa) and a transmembrane β-chain (140 kDa)^1^, encoded by c-Met gene^2^. Under normal physiological conditions, it regulates important cellular processes, such as differentiation, proliferation, cell cycle, motility, and apoptosis. The intracellular portion of c-Met comprises a catalytic tyrosine kinase domain, which contains multifunctional docking sites^3^. Many downstream pathways such as PI3K, MAPK, and STAT3 will be activated, when the HGF binds to the extracellular domains of c-Met, followed by autophosphorylation of tyrosine kinase residues in the catalytic domain^4^. Abnormal activation in the c-Met pathway induces excessive cell proliferation and is related to the development and progression of the neoplastic disease^5^.

With the development of research on the mechanism in drug resistance to cancer cells, the crossing talk between various membrane receptors and c-Met is monitored in tumours and takes part in the resistance to EGFR-tyrosine kinase inhibitors including gefitinib and erlotinib^6^^,^^7^. Continuous angiogenesis is a key mechanism of tumour growth. Therefore, anti-angiogenesis plays an important role in cancer therapy. VEGF has a strong stimulating effect on the proliferation of endothelial cells through increasing vascular permeability and promoting the formation of tumour blood vessels^8^. It specifically binds to the extracellular domain of VEGFR-2 and is involved in endothelial cell sprouting, migration, vascular permeability, and tumour cell survival. However, VEGFR-2 inhibitors can lead to c-Met-dependent invasion and metastasis of tumour cells by increasing the degree of hypoxia when used alone^9^. While the combination of sunitinib and c-Met inhibitor PF-04217903 inhibits both VEGFR and c-Met pathways, significantly decreases tumour invasion and metastasis^10^^,^^11^. Studies on the multi-target c-Met kinase inhibitors show that it is different from the type I inhibitor which possesses high selectivity. The multi-target c-Met kinase inhibitor binds to the inactive conformation of the kinase and belongs to the type II c-Met kinase inhibitor^12^^,^^13^, which binds to not only c-Met but also VEGFR, FGFR, ALK, EGFR, MAT1R. All these studies suggest that simultaneous inhibition of the two tyrosine kinase receptors results in better anti-tumour effects^14^^,^^15^.

The number of multi-target c-Met inhibitors that entering the clinical stage is small and the skeleton of them is lacking in diversity at present, it is easy to cause over-fitting when building virtual screening models according to the ligand^16^. We established a fragment-based multistage screening method for discovering VEGFR-2 inhibitors in previous work^17^^,^^18^, obtained a novel molecule with a high antineoplastic effect. Not affected by the ligand conditions, the fragment-based theory held that the active pockets of drug targets were made up of multiple sub-pockets, and the fragments are units that combine with these sub-pockets. Finding these fragments and linking them together often led to higher active compounds^19^^,^^20^^,^^21^. In this study, we intended to use this method to establish a virtual screening model for discovering multi-target c-Met inhibitors, increasing the structural diversity of these inhibitors.

## 2. Results and discussions

### 2.1. Structural analysis and site map of the binding pocket

**Figure 1.**
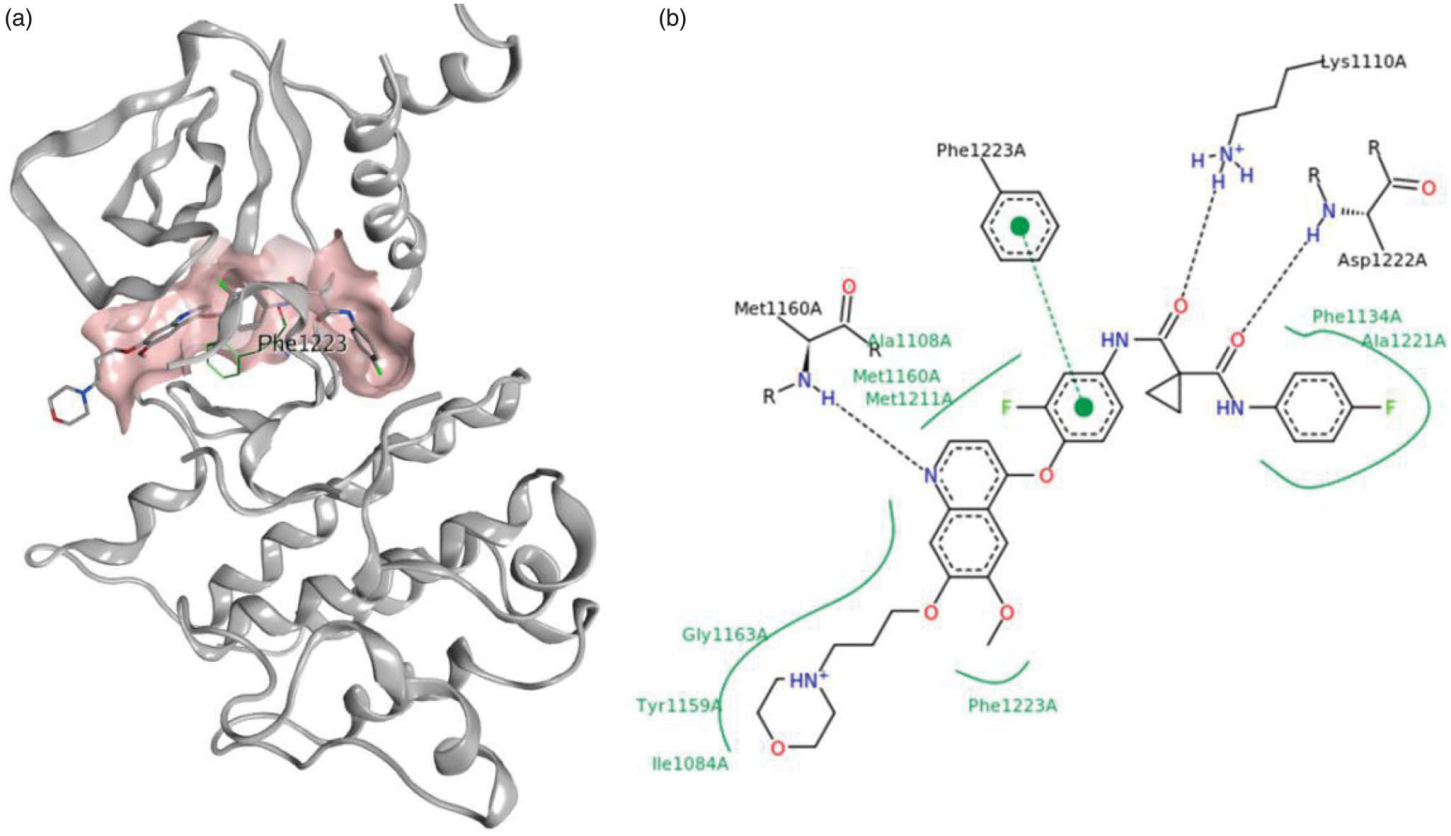
Structure of the kinase domain of c-Met bind to GSK1363089, (a) 3D-view image; (b) pose-view image.

The crystal structures of c-Met and VEGFR-2 kinase are available in the Protein Data Bank (PDB), both of them are in their inactive conformations (PDB IDs: 3LQ8 and 3VHE)^22^^,^^23^. The ATP-binding inactive pocket of c-Met is generally divided into a back pocket behind the gatekeeper (c-Met: Phe1223), and a front pocket within the solvent-accessible area (Figure 1(a)). The back pocket is the unique pocket of the inactive conformation of tyrosine kinase, namely the DFG-out site. The individual pockets are constructed by several amino acids, the surrounding amino acids of each pocket impart distinct properties. The back pocket in c-Met is characterised as a hydrophobic pocket due to the surrounding hydrophobic amino acids: Phe1134, Leu1195, and Phe1200 (Figure 1(b)). Meanwhile, the front-pocket is located in a solvent-accessible area and is surrounded by Ile1084, Lys1161 and His1162 (Figure 1(b)). Analysis of protein-ligand interaction of c-Met inhibitors (exemplified by GSK1363089 in c-Met) showed that the common interaction patterns include: a conserved hydrogen bond with the backbone of Asp1222, an arene–arene stacking interaction with the side chain of Phe1223.

### 2.2. Fragment-based drug design

#### 2.2.1. Fragment growth based on aromatic acyl hydrazine

**Figure 2.**
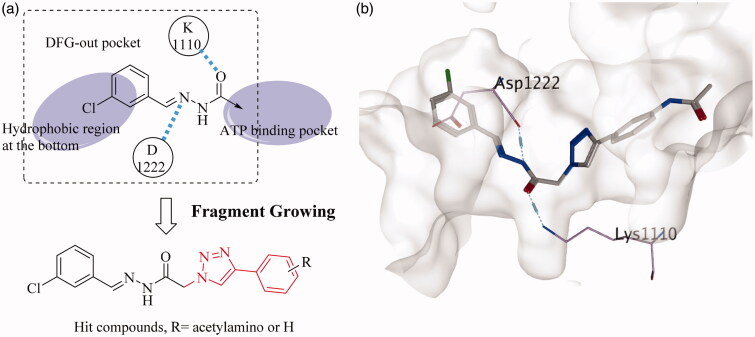
(a) Fragment growing based virtual screening; (b) the hit compound docked into c-Met.

For non-competitive inhibitors combining with the DFG-out site of tyrosine kinases, an ASP and a Lys around gatekeeper are key residues^24^^,^^25^. In our previous work, we found that the *trans*-acylhydrazine can also form hydrogen bonds with Asp1110 and Lys1222 of c-Met. Given that the hydrophobic residues were in the back pocket, the aromatic acyl hydrazine was used as the initial fragment (Figure 2(a)). Then the fragment growth method in MOE was used to generate novel molecules. The newly generated small molecules were then filtered by the screening model of VEGFR-2. Finally, a novel skeleton that linked a phenyl group to the aromatic acyl hydrazine with a triazole fragment was achieved. It was worth noting that a series of small molecules with large substituents on the phenyl group were abandoned by the second model, which indicated that large substituents were not beneficial for increasing the inhibitory activity of small molecules against VEGFR-2.

#### 2.2.2. Docking

The hit compound was docked into the inactive conformation of c-Met (Figure 2(b)). The halogenated aromatic ring was in the hydrophobic region at the bottom of the pocket and the *trans*-acylhydrazine fragment formed two hydrogen bonds with Asp1110 and Lys1222, respectively. On the basis of the new skeleton, the halogen atom at the tail of the novel compound was further changed, exploring the approach to increase the activity of this small molecule. Owing to the large substituents on the phenyl group seem to play an opposite role in the c-Met and VEGFR-2 inhibitory activities of the small molecule, an acetylamino group was taken into account as a compromise.

### 2.3. Chemistry

**Scheme 1.**
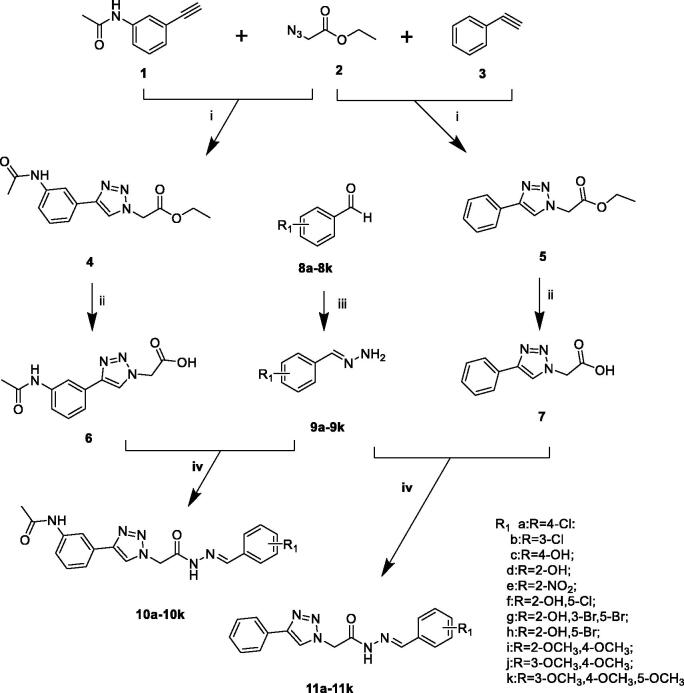
Reagents and conditions: (i) CuSO_4_, Vitamin C, EtOH/H_2_O, 40 °C, 5–10 h; (ii) NaOH, 6 h, HCl; (iii) hydrazine hydrate, rt, 5 h; (iv) EDCI, HOBt, Et_3_N, 35 °C, 8–10 h.

The compounds (**9a–9k**, **10a–10k**, **11a–11k**) were achieved through the route as illustrated in Scheme 1. *N*-(3-ethynylphenyl)acetamide (**1**) was prepared by 3-ethynylaniline and acetic anhydride. The important intermediates (**4**, **5**) were obtained by the corresponding aryl alkyne (**1**, **3**) and ethyl 2-azidoacetate (**2**). Compounds **4** and **5** were subjected to hydrolysis to give intermediates **6** and **7**. The preparation of different (*E*)-benzylidenehydrazine (**9a–9k**) involved benzaldehyde derivatives reaction with hydrazine hydrate. The intermediates (**6**, **7**) were treated by EDCI and HOBt. Then different (*E*)-benzylidenehydrazine (**9a–9k**) and Et_3_N were added with stirring to give the compounds **10a–10k**, **11a–11k**.

In the previously designed route, we used intermediate **5** reacted with hydrazine hydrate and then form the target compounds with benzaldehyde derivatives. We found an interesting phenomenon during the previous synthetic route exploration. There will be *cis***–***trans* isomers generated in this process. So we improved the synthetic route to get the pure target compounds (Scheme 1).

The difficulties in the synthesis of the target compounds (**10a–10k**, **11a–11k**) lie in the *cis***–***trans* isomerisation of the acyl hydrazine fragment. To synthesise a pure single conformation is the focus of synthesis. It was found that acyl hydrazine fragment synthesised by click chemistry link to another fragment results in the appearance of *cis***–***trans* isomers due to the direction of the linkage. The mixture is difficult to separate according to the previous synthesis studies conducted by our group. The yields of the *cis* and *trans* configurations were equal and were confirmed in nuclear magnetics. This was also verified in the study of *cis***–***trans* isomers by Jian Wu^26^, Mohammad Sayed Alam^27^. In response to the above problems, we improved the synthetic route, the benzaldehyde derivatives were used to form the (*E*)-benzylidenehydrazine derivatives fragment with hydrating hydrazine. For another fragment, phenylacetylene and 3-ethynylaniline were used as raw materials and obtained by click chemistry and hydrolysis. The new synthetic method solved the problem of generating *cis***–***trans* isomers, simplified the synthetic method, reduced the synthetic steps and has great reference value for the synthesis and purification of *cis***–***trans* isomers.

### 2.4. QSAR study

The capacity of the novel compounds for inhibiting c-Met activity was evaluated using a cell-free assay. The evaluation results are summarised in Table 1.

**Table 1.** Novel compounds and their activities against c-Met.


Compound	R_1_	R_2_	R_3_	R_4_	R_5_	IC_50_(nM)
						c-Met
**11a**	H	H	H	Cl	H	–^a^
**11c**	H	H	H	OH	H	5681.98
**11d**	H	OH	H	H	H	–
**11e**	H	NO_2_	H	H	H	4276.61
**11g**	H	OH	Br	H	Br	–
**11h**	H	OH	H	H	Br	11.93
**11j**	H	H	OCH_3_	OCH_3_	H	3076.1
**11k**	H	H	OCH_3_	OCH_3_	OCH_3_	–
**10a**	NHCOCH_3_	H	H	Cl	H	1.70
**10c**	NHCOCH_3_	H	H	OH	H	11184.1
**10d**	NHCOCH_3_	OH	H	H	H	266.01
**10e**	NHCOCH_3_	NO_2_	H	H	H	5857.3
**10g**	NHCOCH_3_	OH	Br	H	Br	8933.14
**10h**	NHCOCH_3_	OH	H	H	Br	–
**10i**	NHCOCH_3_	OCH_3_	H	OCH_3_	H	1.73
**10j**	NHCOCH_3_	H	OCH_3_	OCH_3_	H	5.42
**10k**	NHCOCH_3_	H	OCH_3_	OCH_3_	OCH_3_	91.52
GSK1363089						0.6

^a^ –No inhibitory effect.

#### 2.4.1. PCA-based small molecule descriptor selection

To find out the molecules with the optimal structure, a 3D-QSAR study on the derivatives was carried out using the molecular descriptors method in Molecular Operation Environment System (MOE; Chemical Computing Group Inc., Montreal, QC, Canada). Based on the molecular model, which showed a significant correlation with the experimental IC_50_ activity data, further improvement of this correlation was analysed by molecular descriptors. The available molecular descriptors implemented in MOE were tested. As a result, the molecular descriptors were used to analyse the structure-activity relationship of each series. To reduce the model noise and eliminate the interaction between descriptors, the PCA-based small molecule descriptor method was adopted. According to the significant correlation with IC_50_, 10 descriptors were screened out from 379 descriptors by the PCA method (RMSE = 22124.5 *R*^2^ = 0.746558). Target compounds correlation coefficients are shown in Table 2. After calculation, the normalised linear model displayed significant relation to descriptors. The following equation showed the generated QSAR model that included eleven descriptors (AM1_E_, AM1_Eele_, MNDO_E_, PM3_E_, PM3_Eele_, pmi, pmi2, pmi3, pmiX, pmiY) to predict IC_50_/SD values of c-Met inhibitors.

$$\mathrm{QSAR}:\;\frac{{\mathrm{Pred}}_{\mathrm{IC}50}}{\mathrm{SD}(\mathrm{IC}50)}=6.20846-16.95238*\frac{\mathrm{PM}3_{\mathrm{Eele}}}{\mathrm{SD}(\mathrm{PM}3_{\mathrm{Eele}})}+15.93245*\frac{\mathrm{AM}1_{\mathrm{Eele}}}{\mathrm{SD}(\mathrm{AM}1_{\mathrm{Eele}})}+1.14920*\frac{{\mathrm{MNDO}}_{E}}{\mathrm{SD}({\mathrm{MNDO}}_{E})}-0.93352*\frac{\mathrm{pmiY}}{\mathrm{SD}(\mathrm{pmiY})}-5.46574*\frac{\mathrm{PM}3_{E}}{\mathrm{SD}(\mathrm{PM}3_{E})}+0.76667*\frac{\mathrm{pmiX}}{\mathrm{SD}(\mathrm{pmiX})}+4.91929*\frac{\mathrm{AM}1_{E}}{\mathrm{SD}(\mathrm{AM}1_{E})}-0.03510*\frac{\mathrm{pmi}}{\mathrm{SD}(\mathrm{pmi})}-0.21423*\frac{\mathrm{pmi}2}{\mathrm{SD}(\mathrm{pmi}2)}-0.03081*\frac{\mathrm{pmi}3}{\mathrm{SD}(\mathrm{pmi}3)}$$

**Table 2.** Descriptors and relative importance of descriptors.

Descriptors	Relative importance of descriptors
AM1_Eele_	0.904317
PM3_E_	0.500991
AM1_E_	0.281605
PM3_Eele_	0.258684
pmiY	0.122461
pmiX	0.071553
pmi3	0.041214
pmi2	0.032288
MNDO_E_	0.017582
pmi	0.017462

In the QSAR model above, AM1_E_, MNDO_E_, and PM3_E_ were used to describe total energy (molecular dynamic kinetic energy in space, the energy of the electrons of a molecule, intramolecular energy, vibrational energy between atoms in a molecule, the energy of the molecule rotating around the centre of mass); AM1_Eele_ and PM3 _Eele_ represented electronic energy; pmi, pmi2, pmi3, pmiX and pmiY showed principal moment of inertia (measurement of inertia of a rigid body rotating around an axis). The magnitude of the value indicated its correlation with IC_50_. According to the QSAR model, the activity of the compounds was mainly related to electronic energy (AM1_Eele_ and PM3_Eele_).

#### 2.4.2. QSAR suggestion

**Figure 3.**
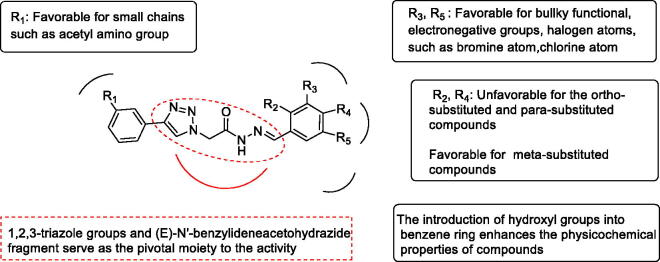
Important structural requirements of (*E*)-*N'*-benzylidene hydrazides by means of the ligand based 3D-QSAR.

After analysis of the contribution of the substituents to the activities by MOE, a QSAR suggestion was given as a set of compounds with more potential activities. The compounds in the QSAR suggestion set combined the advantages of the synthesised compounds and discarded their disadvantages. The results of the QSAR study are shown in Figure 3. The activities of the compounds in series **10** were better than that of series **11**. For the compounds in series **11**, the substituents on the benzene ring preferred for halogen atoms, such as bromine atom. This could be explained by high total energy and electronic energy of compounds that bromine atom brought. While the introduction of hydroxy group reduced the activities of the compounds. As a result of the principal moment of inertia, the activities of the meta-substituted compounds were better than that of the *ortho*-substituted and *para*-substituted compounds. However, the activity of the compound **11h** was superior to **11g**, prompting that it could be dominated by the increased total energy rather than the principal moment of inertia. Therefore, the QSAR model suggested *meta*-chloro substitution (**11b**). For the compounds in series **10**, the contribution of *ortho*-substituents was uncertain. However, the combination of hydroxyl and chlorine atoms could make the molecule have better physicochemical properties since the QSAR suggestion gave **10f**, **11f**, and **11i**. The compound **10i**, **10j**, and **10k** substituted by methoxy showed different inhibitory effects. It may due to the different position and number of substituents brought the change of compounds’ energy and moment of inertia.

The virtual screening model of VEGFR-2 was used to screen the QSAR Suggestion, five molecules were screened out. Followed by synthesising and detecting their kinase activities against c-Met and VEGFR-2, respectively, **11b** and **10f** showed better inhibitory activity than the others as shown in Table 3. It was consistent with the QSAR predicted results. Unfortunately, compound **10b** did not exhibited expected activity against VEGFR-2, which may be related to acetyl substitution. It was not accidental that molecules with activity against c-Met can also inhibit VEGFR-2, the multi-target inhibitors against c-Met and VEGFR-2 have a physiological basis. Both of the proteins belong to the family of tyrosine kinase, which catalyse the phosphorylation of tyrosine. The similar functions make them possess similar active sites. While subtle differences may make small molecules show different activities against the two kinases. So we superimposed the two kinases in three dimensions to explore the reason.

**Table 3.** The inhibitory activities of the five compounds against VEGFR-2 and c-Met.

Name	R_1_	R_2_	R_3_	R_4_	R_5_	VEGFR-2 (nM)	c-Met (nM)
**11b**	H	H	Cl	H	H	25.34	3.41
**10b**	NHCOCH_3_	H	Cl	H	H	–^a^	0.37
**11f**	H	OH	H	H	Cl	9855.76	–
**10f**	NHCOCH_3_	OH	H	H	Cl	139.44	1.19
**11i**	H	OCH_3_	H	OCH_3_	H	849.27	–

^a^ –No inhibitory effect.

**Figure 4.**
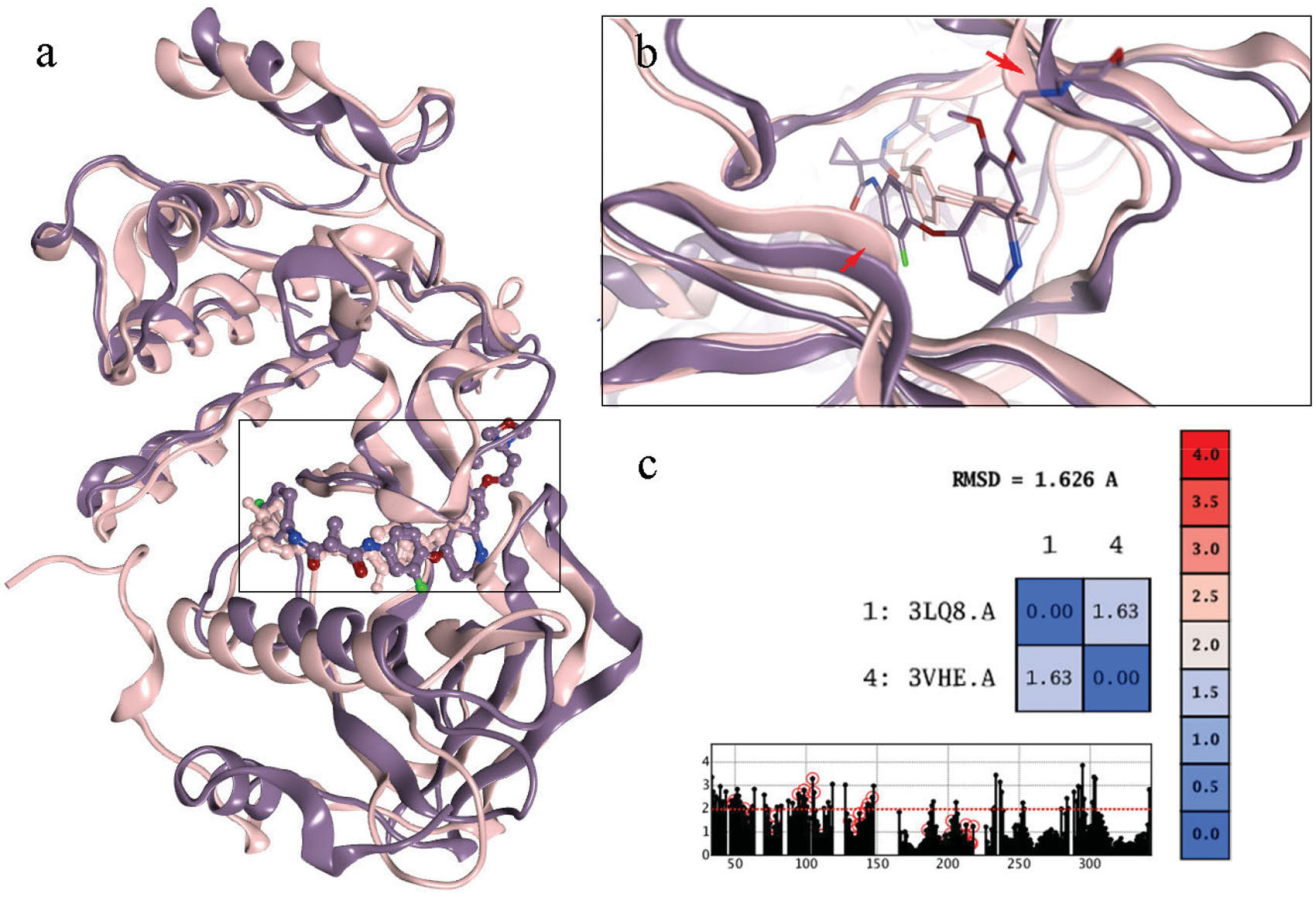
(a) The superposing of c-Met and VEGFR-2 kinase; (b) the active sites of the two kinases; (c) the RMSD value of the superposing conformation.

The structures of c-Met and VEGFR-2 were superposed in 3D space as shown in Figure 4(a). The RMSD results of the superposition were 1.63 Å, indicating that the similarity of the two conformations was very high. Moreover, the back pockets in the two kinases, named the DFG out site, possessed higher similarity (Figure 4(c), the residues marked with red circle). This also provides the basis for the discovery of dual target drugs. However, when the active site was amplified, the difference between the two pockets was detected. The opening of c-Met pocket was wide, which can accommodate small molecules. So the inhibitor of c-Met was longer. While the opening of VEGFR-2 pocket was more narrow that it could not accommodate any group (Figure 4(b), the ribbons marked with red arrows). So the inhibitor of VEGFR-2 was not as long as that of c-Met. So it was suggested that the opening of VEGFR-2 pocket could not accommodate the acetylamino group, causing compound **10b** to show promising inhibitory activity against c-Met but no activity against VEGFR-2.

## 3. Experimental

### 3.1. Synthesis and characterisation of novel compounds

For all target compounds, ^1^H-NMR and ^13^C-NMR spectra were recorded on a Varian NMR spectrometer operating at 600 MHz for ^1^H, and 150 MHz for ^13^C. All chemical shifts were measured in DMSO-d_6_ as solvents. All chemicals were purchased from Sinoreagent Chemical Reagent (Beijing, China) and were used as received, unless stated otherwise. Analytical TLC is performed on silica gel 60 F254 plates (Qingdao Haiyang Chemical Company, Qingdao, Shandong, China) and visualised by UV. Flash column chromatography is performed on gel 60 (40–63 mm) (Qingdao Haiyang Chemical Company, Qingdao, Shandong, China). Melting points were determined with an Electro thermal melting point apparatus, are uncorrected. ^1^H-NMR and ^13^C-NMR of all the target compounds are described in Supplementary material.

General procedure for the synthesis of the ethyl 2–(4-(3-acetamidophenyl)-1H-1, 2, 3-triazol-1-yl) acetate (**4**) and ethyl 2–(4-phenyl-1H-1, 2, 3-triazol-1-yl) acetate (**5**):

A mixture of *N*-(3-ethynylphenyl)acetamide (**1**) (5 g, 31.41 mmol) and ethanol (CH_3_CH_2_OH) (20 ml) was stirred at 40 °C for 15 min. The water solution of copper sulphate pentahydrate (0.3 g) and ascorbic acid (0.3 g) was added and stirred for 5 more minutes. Then ethyl 2-azidoacetate (**2**) (4.87 g, 37.69 mmol) was added. The reaction was stirred at 40 °C for 8 h and monitored by TLC. After completion of the reaction, the solvent was removed in vacuum. Adding water and ethyl acetate, the organic phase was separated and dried over anhydrous sodium sulphate. The solvent was removed in vacuum and the residue was purified by silica gel column to give the pure compound (**4**), as a yellow solid.

Compound **5** was prepared with the same method using ethynylbenzene (**3**), to give pure compound as a pale yellow oily liquid.

General procedure for the synthesis of the 2-(4-(3-acetamidophenyl)-1*H*-1, 2, 3-triazol-1-yl) acetic acid (**6**) and 2-(4-phenyl-1*H*-1, 2, 3-triazol-1-yl) acetic acid (**7**):

To a solution of ethyl 2-(4-(3-acetamidophenyl)-1*H*-1, 2, 3-triazol-1-yl) acetate (**4**) (6 g, 20.81 mmol), sodium hydroxide (3.33 g, 83.24 mmol) in 30 ml water was added. The reaction solution was stirred at 40 °C for 5 h. After completion of the reaction, dilute hydrochloric acid was slowly added to adjust the solution pH to 2. The obtained solid was filtered and dried to give the compound 2-(4-(3-acetamidophenyl)-1H-1, 2, 3-triazol-1-yl) acetic acid (**6**) as a light yellow solid.

Compound **7** was prepared with the same method by using corresponding raw materials **5**.

General procedure for the synthesis of the (*E*)-benzylidenehydrazine derivatives (**9a–9k**):

A solution of 80% hydrazine hydrate (8.9 g, 177.85 mmol) was added dropwise to 4-chlorobenzaldehyde (**8a**) (5.0 g, 35.57 mmol) in 10 ml water. The reaction mixture was stirred at room temperature for 5 h and monitored by TLC. After completion of the reaction, the reaction mixture was poured into 50 ml water and stirred for 20 min. The reaction mixture was filtered off. Filter cake was washed with water, and dried to give the compound **9a** as a white solid, yield: 81.27%.

Compounds **9b** to **9k** were prepared with the same method by using corresponding raw materials **8b** to **8k**.

General procedure for preparation of target compounds (**10a–10k, 11a–11k**):

A solution of 2-(4-(3-acetamidophenyl)-1*H*-1, 2, 3-triazol-1-yl) acetic acid (**6**) (2.46 mmol) and 1-hydroxybenzotriazole (2.58 mmol) in 20 ml DMF was stirred at −5 °C for 20 min. Then 1-ethyl-3-(3-dimethylaminopropyl) carbodiimide hydrochloride (2.58 mmol) was added and stirred for 15 min. The reaction mixture was stirred in 40 °C oil bath and compounds **9a–9k** (2.58 mmol), triethylamine(7.38 mmol) were added. The reaction was stirred at 40 °C for 8–10 h and monitored by TLC. After completion of the reaction, slowly pour the liquid into 150 ml water. After adding ethyl acetate in the mixture, the organic phase was separated and dried over anhydrous sodium sulphate. The solvent was removed in vacuum. The residue was purified by silica gel column to give the target compounds (**10a–10k**).

Compounds **11a–11k** were prepared with the same method by using the corresponding intermediate **7**.

#### 3.1.1. (*E*)-2-(4-(3-acetamidophenyl)-1*H*-1, 2, 3-triazol-1-yl)-*N'*-(4-chlorobenzylidene) (10a)

A white solid, yield: 61.24%. Mp:234.6–234.9 °C ^1^H NMR (600 MHz, DMSO-d_6_) *δ* 11.96 (s, 1H, –N–H), 10.07 (s, 1H, –N–H), 8.50 (s, 1H, –N–CH=), 8.16 (s, 1H, Ar–H), 8.07 (s, 1H, –N = CH–), 7.81 (d, *J* = 8.5 Hz, 2H, Ar–H), 7.58 (d, *J* = 7.4 Hz, 1H, Ar–H), 7.53 (d, *J* = 8.5 Hz, 2H, Ar–H), 7.49 (d, *J* = 7.4 Hz, 1H, Ar–H), 7.38 (t, *J* = 7.9 Hz, 1H, Ar–H), 5.77 (s, 2H, CH_2_), 2.08 (s, 3H, CH_3_). ^13 ^C NMR (150 MHz, DMSO-d_6_) *δ* 167.84, 166.92, 145.49, 142.65, 139.31, 134.00, 132.21, 130.58, 128.73, 128.32, 128.13, 122.59, 119.35, 117.79, 114.91, 50.12, 23.48.

#### 3.1.2. (*E*)-2-(4-(3-acetamidophenyl)-1*H*-1, 2, 3-triazol-1-yl)-*N'*-(3-chlorobenzylidene) (10b)

A white solid, yield: 57.21%. Mp:255.8–256.3 °C ^1^H NMR (600 MHz, DMSO-d_6_) *δ* 12.01 (s, 1H, –N–H), 10.09 (s, 1H, –N–H), 8.51 (s, 1H, –N–CH=), 8.17 (s, 1H, Ar–H), 8.07 (s, 1H, –N = CH–), 7.94 (s, 1H, Ar–H), 7.89 (s, 1H, Ar–H), 7.86 (d, *J* = 7.6 Hz, 1H, Ar–H), 7.59 (d, *J* = 7.6 Hz, 1H, Ar–H), 7.56 (d, *J* = 7.7 Hz, 1H, Ar–H), 7.37 (m, 2H, Ar–H), 5.80 (s, 2H, CH_2_), 2.09 (s, 3H, CH_3_). ^13 ^C NMR (150 MHz, DMSO-d_6_) *δ* 168.91, 168.13, 161.12, 146.56, 143.38, 140.38, 136.25, 134.19, 131.64, 131.34, 130.24, 129.79, 128.34, 127.40, 123.65, 120.43, 115.99, 51.26, 24.55.

#### 3.1.3. (*E*)-2-(4-(3-acetamidophenyl)-1*H*-1, 2, 3-triazol-1-yl)-*N'*-(4-hydroxybenzylidene) acetohydrazide (10c)

A white solid, yield: 52.11%. Mp:260.0–260.8 °C ^1^H NMR (600 MHz, DMSO-d_6_) *δ* 11.70 (s, 1H, –N–H), 10.07 (s, 1H, –O–H), 10.00 (s, 1H, –N–H), 8.50 (s, 1H, –N–CH=), 8.16 (s, 1H, Ar–H), 7.97 (s, 1H, –N = CH–), 7.60 (d, *J* = 8.7 Hz, 2H, Ar–H), 7.56 (d, *J* = 8.7 Hz, 1H, Ar–H), 7.49 (d, *J* = 7.6 Hz, 1H, Ar–H), 7.37 (t, *J* = 7.9 Hz, 1H, Ar–H), 6.85 (d, *J* = 8.7 Hz, 2H, Ar–H), 5.71 (s, 2H, CH_2_), 2.08 (s, 3H, CH_3_). ^13^C NMR (150 MHz, DMSO-d_6_) *δ* 168.91, 167.45, 159.94, 148.69, 146.52, 145.29, 140.37, 131.68, 129.80, 129.52, 129.28, 125.31, 123.68, 120.42, 118.85, 116.18, 115.97, 51.14, 24.55.

#### 3.1.4. (*E*)-2-(4-(3-acetamidophenyl)-1*H*-1,2,3-triazol-1-yl)-*N'*-(2-hydroxybenzylidene) acetohydrazide (10d)

A white solid, yield: 53.24%. Mp:253.8–254.2 °C ^1^H NMR (600 MHz, DMSO-d_6_) *δ* 11.81 (s, 1H, –N–H), 10.07 (s, 2H, –N–H, –O–H), 8.50 (s, 1H, –N–CH=), 8.39 (s, 1H, –N = CH–), 8.16 (s, 1H, Ar–H), 7.58 (d, *J* = 5.5 Hz, 1H, Ar–H), 7.49 (d, *J* = 7.4 Hz, 1H, Ar–H), 7.38 (t, *J* = 7.9 Hz, 1H, Ar–H), 7.27 (t, *J* = 7.0 Hz, 1H, Ar–H), 6.92 (m, 2H, Ar–H), 6.88 (t, *J* = 7.5 Hz, 1H, Ar–H), 5.72 (s, 2H, CH_2_), 2.08 (s, 3H, CH_3_). ^13^C NMR (150 MHz, DMSO-d_6_) δ 168.91, 167.57, 162.48, 156.95, 148.23, 146.53, 142.15, 140.38, 131.89, 129.80, 126.69, 123.67, 120.49, 119.89, 116.84, 116.63, 115.97, 51.18, 24.55.

#### 3.1.5. (*E*)-2-(4-(3-acetamidophenyl)-1*H*-1,2,3-triazol-1-yl)-*N'*-(2-nitrobenzylidene)acetohydrazide (10e)

A yellow solid, yield: 45.21%. Mp: 233.2–233.8 °C ^1^H NMR (600 MHz, DMSO-d_6_) *δ* 12.17 (s, 1H, –N–H), 10.07 (s, 1H, –N–H), 8.51 (s, 1H, –N–CH=), 8.47 (s, 1H, –N = CH–), 8.17 (s, 1H, Ar–H), 7.82 (t, *J* = 7.5 Hz, 1H, Ar–H), 7.69 (t, *J* = 7.3 Hz, 1H, Ar–H), 7.59 (m, 2H, Ar–H), 7.50 (d, *J* = 7.4 Hz, 1H, Ar–H), 7.40 – 7.35 (m, 2H, Ar–H), 5.77 (s, 2H, CH_2_), 2.08 (s, 3H, CH_3_). ^13^C NMR (150 MHz, DMSO-d_6_) *δ* 167.85, 167.18, 147.49, 145.54, 139.60, 139.32, 133.04, 130.16, 128.74, 127.94, 127.51, 124.01, 122.59, 121.48, 119.37, 117.81, 114.92, 50.13, 23.49.

#### 3.1.6. (*E*)-2-(4-(3-acetamidophenyl)-1*H*-1,2,3-triazol-1-yl)-*N'*-(5-chloro-2-hydroxybenzylidene) acetohydrazide (10f)

A white solid, yield: 58.29%. Mp:234.6–234.9 °C ^1^H NMR (600 MHz, DMSO-d_6_) *δ* 11.88 (s, 1H, –N–H), 10.44 (s, 1H, –O–H), 10.06 (s, 1H, –N–H), 8.48 (s, 1H, –N–CH=), 8.32 (s, 1H, –N = CH–), 8.15 (s, 1H, Ar–H), 7.58 (d, *J* = 7.5 Hz, 1H, Ar–H), 7.49 (d, *J* = 7.3 Hz, 1H, Ar–H), 7.37 (t, *J* = 7.8 Hz, 1H, Ar–H), 7.09 (s, 1H, Ar–H), 7.01 (d, *J* = 8.8 Hz, 1H, Ar–H), 6.94 (d, *J* = 8.8 Hz, 1H, Ar–H), 5.78 (s, 2H, CH_2_), 2.08 (s, 3H, CH_3_). ^13^C NMR (150 MHz, DMSO-d_6_) *δ* 168.90, 167.90, 161.33, 140.37, 133.21, 131.27, 129.79, 129.09, 128.27, 127.15, 123.78, 123.63, 122.37, 120.46, 118.99, 117.87, 115.97, 51.27, 24.54.

#### 3.1.7. (*E*)-2-(4-(3-acetamidophenyl)-1H-1,2,3-triazol-1-yl)-*N'*-(3,5-dibromo-2-hydroxybenzylidene)acetohydrazide (10g)

A white solid, yield: 62.31%. Mp:237.0–237.5 °C ^1^H NMR (600 MHz, DMSO-d_6_) *δ* 12.63 (s, 1H, –N–H), 12.23 (s, 1H, –O–H), 12.04 (s, 1H, –N–H), 8.56 (s, 1H, –N–CH=), 8.46 (s, 1H, –N = CH–), 8.42 (s, 1H, Ar–H), 8.31 (s, 1H, Ar–H), 7.94 (s, 1H, Ar–H), 7.58 (d, *J* = 8.0 Hz, 1H, Ar–H), 7.50 (d, *J* = 7.2 Hz, 1H, Ar–H), 7.37 (s, 1H, Ar–H), 5.81 (s, 2H, CH_2_), 2.08 (s, 3H, CH_3_). ^13^C NMR (150 MHz, DMSO-d_6_) *δ* 168.89, 163.02, 154.00, 152.70, 147.84, 146.75, 140.38, 136.31, 132.67, 131.49, 129.79, 123.51, 121.35, 120.50, 116.07, 111.77, 111.01, 51.35, 24.54.

#### 3.1.8. (*E*)-2-(4-(3-acetamidophenyl)-1*H*-1,2,3-triazol-1-yl)-*N'*-(5-bromo-2-hydroxybenzylidene) acetohydrazide (10h)

A white solid, yield: 57.64%. Mp:228.9–229.3 °C ^1^H NMR (600 MHz, DMSO-d_6_) *δ* 11.85 (s, 1H, –N–H), 10.39 (s, 1H, –O–H), 10.04 (s, 1H, –N–H), 8.47 (s, 1H, –N–CH=), 8.30 (s, 1H, –N = CH–), 8.14 (s, 1H, Ar–H), 7.95 (s, 1H, Ar–H), 7.57 (d, *J* = 4.9 Hz, 1H, Ar–H), 7.48 (d, *J* = 7.8 Hz, 1H, Ar–H), 7.43–7.34 (m, 3H, Ar–H), 5.77 (s, 2H, CH_2_), 2.07 (s, 3H, CH_3_). ^13^C NMR (150 MHz, DMSO-d_6_) *δ* 168.89, 167.90, 162.78, 156.11, 146.49, 140.37, 140.10, 134.11, 131.68, 129.80, 128.32, 123.63, 122.92, 120.42, 118.87, 115.96, 111.39, 51.27, 24.55.

#### 3.1.9. (*E*)-2-(4-(3-acetamidophenyl)-1*H*-1,2,3-triazol-1-yl)-*N'*-(2,4-dimethoxybenzylidene) acetohydrazide (10i)

A yellow solid, yield: 59.36%. Mp:210.9–211.3 °C ^1^H NMR (600 MHz, DMSO-d_6_) *δ* 10.05 (s, 1H, –N–H), 8.83 (s, 1H, –N–CH=), 8.42 (s, 1H, –N = CH–), 8.16 (s, 1H, Ar–H), 7.91 (d, *J* = 8.6 Hz, 1H, Ar–H), 7.55 (d, *J* = 8.0 Hz, 1H, Ar–H), 7.48 (d, *J* = 7.7 Hz, 1H, Ar–H), 7.36 (t, *J* = 7.9 Hz, 1H, Ar–H), 6.66 (s, 1H, Ar–H), 6.64 (d, *J* = 8.6 Hz, 1H, Ar–H), 5.47 (s, 2H, CH_2_), 3.88 (s, 3H, CH_3_), 3.84 (s, 3H, CH_3_), 2.07 (s, 3H, CH_3_). ^13^C NMR (150 MHz, DMSO-d_6_) *δ* 167.81, 163.92, 162.77, 159.54, 154.92, 145.34, 139.28, 130.64, 128.69, 127.10, 122.60, 119.32, 117.71, 114.85, 114.09, 105.94, 97.63, 55.26, 54.92, 50.05, 23.47.

#### 3.1.10. (*E*)-2-(4-(3-acetamidophenyl)-1*H*-1,2,3-triazol-1-yl)-*N'*-(3,4-dimethoxybenzylidene) acetohydrazide (10j)

A white solid, yield: 54.32%. Mp:230.0–230.6 °C ^1^H NMR (600 MHz, DMSO-d_6_) *δ* 11.80 (s, 1H, –N–H), 10.08 (s, 1H, –N–H), 8.52 (s, 1H, –N–CH=), 8.17 (s, 1H, Ar–H), 8.00 (s, 1H, –N = CH–), 7.59 (d, *J* = 7.7 Hz, 1H, Ar–H), 7.50 (d, *J* = 7.3 Hz, 1H, Ar–H), 7.41 (s, 1H, Ar–H), 7.38 (t, *J* = 7.9 Hz, 1H, Ar–H), 7.23 (d, *J* = 6.7 Hz, 1H, Ar–H), 7.02 (d, *J* = 8.2 Hz, 1H, Ar–H), 5.77 (s, 2H, CH_2_), 3.83 (s, 3H, CH_3_), 3.81 (s, 3H, CH_3_), 2.08 (s, 3H, CH_3_). ^13^C NMR (150 MHz, DMSO-d_6_) *δ* 167.86, 166.61, 150.12, 148.46, 145.50, 143.99, 139.32, 130.62, 128.74, 125.98, 122.60, 121.01, 119.37, 117.79, 114.91, 110.82, 107.89, 54.95, 54.90, 50.20, 23.48.

#### 3.1.11. (*E*)-2-(4-(3-acetamidophenyl)-1*H*-1,2,3-triazol-1-yl)-*N'*-(3,4,5-trimethoxybenzylidene) acetohydrazide (10k)

A yellow solid, yield: 58.21%. Mp:210.2–212.9 °C ^1^H NMR (600 MHz, DMSO-d_6_) *δ* 11.90 (s, 1H, –N–H), 10.05 (s, 1H, –N–H), 8.51 (s, 1H, –N–CH=), 8.16 (s, 1H, Ar–H), 7.99 (s, 1H, –N = CH–), 7.58 (d, *J* = 8.0 Hz, 1H, Ar–H), 7.49 (d, *J* = 7.7 Hz, 1H, Ar–H), 7.37 (t, *J* = 7.9 Hz, 1H, Ar–H), 7.08 (s, 2H, Ar–H), 5.78 (s, 2H, CH_2_), 3.85 (d, 9H, CH_3_), 2.08 (s, 3H, CH_3_). ^13^C NMR (150 MHz, DMSO-d_6_) *δ* 168.89, 167.87, 162.76, 161.67, 153.66, 146.58, 144.82, 140.38, 139.71, 131.68, 129.77, 123.63, 120.42, 118.88, 116.03, 106.09, 104.84, 60.58, 56.46, 36.23, 31.22, 24.51.

#### 3.1.12. (*E*)-*N'*-(4-chlorobenzylidene)-2-(4-phenyl-1*H*-1,2,3-triazol-1-yl)acetohydrazide (11a)

A white solid, yield: 66.72%. Mp: 259.0–259.6 °C. ^1^H NMR (600 MHz, DMSO-d_6_) *δ* 11.94 (s, 1H, –N–H), 8.56 (s, 1H, –N–CH=), 8.07 (s, 1H, –N = CH–), 7.87 (d, *J* = 7.1 Hz, 2H, Ar–H), 7.80 (d, *J* = 8.5 Hz, 2H, Ar–H), 7.52 (d, *J* = 8.5 Hz, 2H, Ar–H), 7.47 (t, *J* = 7.7 Hz, 2H, Ar–H), 7.35 (t, *J* = 7.4 Hz, 1H, Ar–H), 5.76 (s, 2H, CH_2_). ^13^C NMR (150 MHz, DMSO-d_6_) *δ* 167.94, 162.68, 147.15, 146.62, 143.75, 135.07, 133.29, 131.27, 129.41, 129.21, 128.29, 125.59, 123.64, 51.16.

#### 3.1.13. (*E*)-*N'*-(3-chlorobenzylidene)-2-(4-phenyl-1*H*-1,2,3-triazol-1-yl)acetohydrazide (11b)

A white solid, yield: 63.45%. Mp: 259.0–259.8 °C. ^1^H NMR (600 MHz, DMSO-d_6_) *δ* 11.99 (s, 1H, –N–H), 8.55 (s, 1H, –N–CH=), 8.05 (s, 1H, –N = CH–), 7.87 (m, 3H, Ar–H), 7.73–7.69 (t, 1H, Ar–H), 7.51–7.44 (m, 5H, Ar–H), 7.34 (t, *J* = 7.4 Hz, 1H, Ar–H), 5.77 (s, 2H, CH_2_). ^13^C NMR (150 MHz, DMSO-d_6_) *δ* 168.25, 146.58, 143.25, 136.71, 134.19, 131.15, 130.14, 129.41, 128.27, 126.63, 126.44, 125.58, 123.63, 51.26.

#### 3.1.14. (*E*)-*N'*-(4-hydroxybenzylidene)-2-(4-phenyl-1*H*-1,2,3-triazol-1-yl)acetohydrazide (11c)

A white solid, yield: 58.23%. Mp: 234.0–234.5 °C. ^1^H NMR (600 MHz, DMSO-d_6_) *δ* 11.68 (s, 1H, –N–H), 9.94 (s, 1H, –O–H), 8.56 (s, 1H, –N–CH=), 7.98 (s, 1H, –N = CH–), 7.89 (d, 2H, Ar–H), 7.60 (d, *J* = 7.4 Hz, 2H, Ar–H), 7.49–7.34 (m, 3H, Ar–H), 6.85 (d, *J* = 6.7 Hz, 2H, Ar–H), 5.71 (s, 2H, CH_2_). ^13^C NMR (150 MHz, DMSO-d_6_) *δ* 167.41, 159.94, 146.60, 145.33, 131.30, 129.40, 129.29, 128.27, 125.59, 125.34, 123.67, 116.18, 51.13.

#### 3.1.15. (*E*)-*N'*-(2-hydroxybenzylidene)-2-(4-phenyl-1*H*-1,2,3-triazol-1-yl)acetohydrazide (11d)

A white solid, yield: 63.45%. Mp: 239.0–239.6 °C. ^1^H NMR (600 MHz, DMSO-d_6_) *δ* 11.78 (s, 1H, –N–H), 10.04 (s, 1H, –O–H), 8.55 (s, 1H, –N–CH=), 8.38 (s, 1H, –N = CH–), 7.90–7.85 (m, 3H, Ar–H), 7.80 (d, *J* = 6.3 Hz, 1H, Ar–H), 7.46 (m, 3H, Ar–H), 7.35 (d, *J* = 7.5 Hz, 1H, Ar–H), 7.29–7.24 (t, *J* = 7.8 Hz,1H, Ar–H), 5.73 (s, 2H, CH_2_). ^13^C NMR (150 MHz, DMSO-d_6_) *δ* 167.53, 156.95, 146.59, 142.22, 131.88, 131.30, 129.41, 128.28, 126.74, 125.59, 123.66, 120.51, 119.89, 116.64, 51.15.

#### 3.1.16. (*E*)-*N'*-(2-nitrobenzylidene)-2-(4-phenyl-1*H*-1,2,3-triazol-1-yl)acetohydrazide (11e)

A light yellow solid, yield: 53.14%. Mp: 225.2–225.6 °C. ^1^H NMR (600 MHz, DMSO-d_6_) *δ* 12.15 (s, 1H, –N–H), 8.56 (s, 1H, –N–CH=), 8.47 (s, 1H, –N = CH–), 7.88 (d, *J* = 6.5 Hz, 2H, Ar–H), 7.81 (t, *J* = 7.6 Hz, 1H, Ar–H), 7.69 (t, *J* = 7.8 Hz, 1H, Ar–H), 7.49–7.45 (m, 3H, Ar–H), 7.35 (t, *J* = 7.3 Hz, 2H, Ar–H), 5.77 (s, 2H, CH_2_). ^13 ^C NMR (150 MHz, DMSO-d_6_) *δ* 168.20, 148.57, 146.88, 146.67, 140.65, 134.08, 131.22, 129.41, 129.02, 128.45, 125.65, 125.05, 123.64, 123.22, 53.06.

#### 3.1.17. (*E*)-*N'*-(5-chloro-2-hydroxybenzylidene)-2-(4-phenyl-1*H*-1,2,3-triazol-1-yl)acetohydrazide (11f)

A white solid, yield: 48.59%. Mp: 240.0–240.6 °C. ^1^H NMR (600 MHz, DMSO-d_6_) *δ* 11.86 (s, 1H, –N–H), 10.37 (s, 1H, –O–H), 8.54 (s, 1H, –N–CH=), 8.32 (s, 1H, –N = CH–), 7.87 (d, *J* = 7.4 Hz, 2H, Ar–H), 7.83 (d, *J* = 2.6 Hz, 1H, Ar–H), 7.46 (m, 3H, Ar–H), 7.35 (d, *J* = 7.5 Hz, 1H, Ar–H), 7.29 (d, *J* = 8.8 Hz, 1H, Ar–H), 5.77 (s, 2H, CH_2_). ^13^C NMR (150 MHz, DMSO-d_6_) *δ* 167.89, 162.65, 155.69, 146.56, 140.20, 131.29, 129.42, 128.28, 125.57, 125.44, 123.80, 123.64, 122.37, 118.42, 51.27.

#### 3.1.18. (*E*)-*N'*-(3,5-dibromo-2-hydroxybenzylidene)-2-(4-phenyl-1*H*-1,2,3-triazol-1-yl) acetohydrazide (11g)

A light yellow solid, yield: 55.47%. Mp: 231.0–231.5 °C. ^1^H NMR (600 MHz, DMSO-d_6_) *δ* 12.58 (s, 1H, –O–H), 12.02 (s, 1H, –N–H), 8.62 (s, 1H, –N = CH–), 8.52 (s, 1H, –N–CH=), 8.41 (s, 1H, Ar–H), 8.29 (s, 1H, Ar–H), 7.47 (m, 3H, Ar–H), 7.35 (d, *J* = 1.9 Hz, 2H, Ar–H), 5.81 (s, 2H, CH_2_). ^13^C NMR (150 MHz, DMSO-d_6_) *δ* 167.85, 163.01, 147.88, 146.81, 141.75, 136.12, 132.68, 131.11, 129.42, 128.39, 125.65, 125.60, 123.49, 121.34, 51.38.

#### 3.1.19. (*E*)-*N'*-(5-bromo-2-hydroxybenzylidene)-2-(4-phenyl-1*H*-1,2,3-triazol-1-yl)acetohydrazide (11h)

A white solid, yield: 58.55%. Mp: 233.0–233.5 °C. ^1^H NMR (600 MHz, DMSO-d_6_) *δ* 11.85 (s, 1H, –N–H), 10.39 (s, 1H, –O–H), 8.54 (s, 1H, –N–CH=), 8.30 (s, 1H, –N = CH–), 7.95 (s, 1H, Ar–H), 7.87 (m, 2H, Ar–H), 7.46 (m, 3H, Ar–H), 7.41 (d, *J* = 8.7 Hz, 1H, Ar–H), 7.35 (d, *J* = 7.4 Hz, 1H, Ar–H), 5.77 (s, 2H, CH_2_). ^13^C NMR (150 MHz, DMSO-d_6_) *δ* 167.18, 155.49, 145.94, 139.60, 133.45, 130.68, 128.76, 127.62, 125.36, 124.96, 122.96, 122.31, 118.26, 110.76, 50.62.

#### 3.1.20. (*E*)-*N'*-(2,4-dimethoxybenzylidene)-2-(4-phenyl-1H-1,2,3-triazol-1-yl)acetohydrazide (11i)

A yellow solid, yield: 49.52%. Mp: 230.0–230.4 °C. ^1^H NMR (600 MHz, DMSO-d_6_) *δ* 11.79 (s, 1H, –N–H), 8.64 (s, 1H, –N = CH–), 8.57 (s, 1H, –N–CH=), 7.99 (s, 1H, Ar–H), 7.87 (d, *J* = 7.6 Hz, 2H, Ar–H), 7.49 (d, *J* = 1.3 Hz, 1H, Ar–H), 7.46 (d, *J* = 7.7 Hz, 1H, Ar–H), 7.40–7.37 (m, 2H, Ar–H), 7.35 (t, *J* = 7.4 Hz, 1H, Ar–H), 5.76 (s, 2H, CH_2_), 3.82 (s, 6H, CH_3_). ^13^C NMR (150 MHz, DMSO-d_6_) *δ* 163.22, 160.00, 157.87, 155.31, 148.89, 146.34, 133.50, 129.89, 127.55, 122.84, 122.15, 117.82, 114.60, 106.41, 99.36, 98.13, 56.55, 55.73, 55.37.

#### 3.1.21. (*E*)-*N'*-(3,4-dimethoxybenzylidene)-2-(4-phenyl-1H-1,2,3-triazol-1-yl)acetohydrazide (11j)

A white solid, yield: 67.14%. Mp: 232.1–232.5 °C. ^1^H NMR (600 MHz, DMSO-d_6_) *δ* 11.80 (s, 1H, –N–H), 8.65 (s, 1H, –N = CH–), 8.58 (s, 1H, –N–CH=), 8.00 (s, 1H, Ar–H), 7.88 (d, *J* = 7.1 Hz, 2H, Ar–H), 7.47 (m, 3H, Ar–H), 7.41 (d, *J* = 1.7 Hz, 1H, Ar–H), 7.36 (d, *J* = 7.4 Hz, 1H, Ar–H), 5.77 (s, 2H, CH_2_), 3.83 (s, 6H, CH_3_). ^13^C NMR (150 MHz, DMSO-d_6_) *δ* 167.62, 151.23, 149.56, 146.62, 145.09, 131.30, 129.41, 128.28, 127.07, 125.59, 123.64, 122.06, 111.95, 109.08, 56.05, 51.24.

#### 3.1.22. (*E*)-2-(4-phenyl-1*H*-1,2,3-triazol-1-yl)-*N'*-(3,4,5-trimethoxybenzylidene)acetohydrazide (11k)

A white solid, yield: 65.31%. Mp: 233.5–233.8 °C. ^1^H NMR (600 MHz, DMSO-d_6_) *δ* 11.91 (s, 1H, –N–H), 8.66 (s, 1H, –N = CH–), 8.57 (s, 1H, –N–CH=), 7.87 (d, *J* = 7.2 Hz, 2H, Ar–H), 7.47 (m, 2H, Ar–H), 7.35 (t, *J* = 7.2 Hz, 1H, Ar–H), 7.22 (s, 2H, Ar–H), 5.77 (s, 2H, CH_2_), 3.74 (s, 6H, CH_3_), 3.71 (s, 3H, CH_3_). ^13^C NMR (150 MHz, DMSO-d_6_) *δ* 167.86, 161.68, 153.65, 146.62, 144.83, 140.72, 139.71, 131.29, 129.74, 129.42, 128.29, 125.58, 123.64, 106.10, 104.86, 102.78, 100.00, 60.65, 56.43, 51.29.

### 3.2. Cell-free detection of c-Met kinase activity

The compounds inhibitory activities of inhibitors against c-Met were determined using ADP-Glo assay. The reaction buffer was deployed by 50 mM HEPES, pH 7.5, 10 mM MgCl_2_, 0.1 mg/mL BSA, 2 mM DTT and 1% DMSO. The recombinant c-Met kinase was diluted to 2.2 μg/mL using reaction buffer (50 mM HEPES, pH 7.5, 10 mM MgCl_2_, 0.1 mg/mL BSA, 2 mM DTT, 1% DMSO), and ATP was diluted with reaction buffer (10 mM) to 250 μM, the test compounds and positive drug (GSK1363089) were formulated into four concentration gradient solutions (6 × 10^−2 ^M, 6 × 10^−4 ^M, 6 × 10 ^−6 ^M, 6 × 10^−8 ^M). The reaction was started by sequentially adding 2 μL of a ATP solution, 1 μL of a drug solution, and 2 μL of an enzyme solution in 96 wells. The assay was conducted for 1 h at 37 °C before addition of 5 μL ADP-Glo reagent and incubation for 40 min at room temperature. About 10 μL kinase detection reagent was added and incubated for 30 min at room temperature before the luminescence value was measured using a chemiluminescence module of a full-wave length multi-function microplate reader.

### 3.3. Fragment-based drug design

Fragment based drug design was performed using the Add Group to Ligand module in Molecular Operating Environment package (MOE; Chemical Computing Group Inc., Montreal, QC, Canada). The aromatic acyl hydrazine was used as the initial fragment, the hydrogen atom at the head of the acetylhydrazine group was selected as the connection point. During the generation, a filter of Lipinski's rule of five was toggled ON. The novel small molecules were generated based on the shape of the active pocket.

### 3.4. QSAR study

The target compounds database was prepared using compute module in MOE. The preparation involved introduction of every single target compounds using ChemDraw, converted structure to 3D and saved as mol2 formats in Chem3D. Further preparation comprised compounds database generation with importing all mol2 formats and energy minimise in molecule module in compute module using default settings. The IC_50_ activities data were added in another field. Summaries of the relevant datasets employed for generating the QSARs relating the various molecular descriptors to the compounds **10a–10k** and **11a–11k**.

The Regression analysis was performed in QuaSAR module. The QSAR model was performed for compounds **10a–10k** and **11a–11k** using IC_50_ as activity field and PCR method. The RMSE and *R*^2^ values were derived from Quasar fit. The estimated linear model which contained all molecular descriptors was established. The QSAR model was constructed for the **10a–10k** and **11a–11k** compounds with relatively important descriptors.

The database contained compounds structures, IC_50_ values and relatively important descriptors were established. The basic parent structure molecule was input as a guide and scaffolds were calculated as preset. The IC_50_ style was changed into KI/IC_50_ (nM).

### 3.5. Superposing

The PDB files of the two proteins were imported into MOE, and operated in the sequence editing panel. First, the sequences of the two proteins were aligned to ensure the similarity. Then the 3D structures of the two proteins were superposed with each other, which was visible in the main panel of MOE. Finally, the RMSD result was obtained in the protein Superpose RMSD plot panel.

## 4. Conclusion

The difficulty in the synthesis of the target compound (**10a–10k, 11a–11k**) lies in the *cis-trans*isomerizm. After improving the synthetic route, two series of (*E*)-*N'*-benzylidene derivatives were synthesised and evaluated for their inhibitory activities against c-Met. The QSAR results showed that halogens and acetyl of aromatic rings could improve the activity of the compounds against c-Met. However, the compounds with acetyl groups did not perform well in the activity test of VEGFR-2. Nevertheless, we have obtained a highly active type I c-Met inhibitor **10b** and a multi-target c-Met kinase inhibitor **11b**. They could be potential antitumor agents that deserve further research.

## Supplementary Material

Supplemental Material
