# Supplementary material for: Synthesis and biological evaluation of novel (E)-N'-benzylidene hydrazides as novel c-Met inhibitors through fragment based virtual screening
Source: J Enzyme Inhib Med Chem. 2020 Jan 6;35(1):468–77. doi: 10.1080/14756366.2019.1702655 (PMC6968643; doi:10.1080/14756366.2019.1702655)
Supplement: Supplemental Material [file IENZ_A_1702655_SM9022.pdf]

Supplementary document

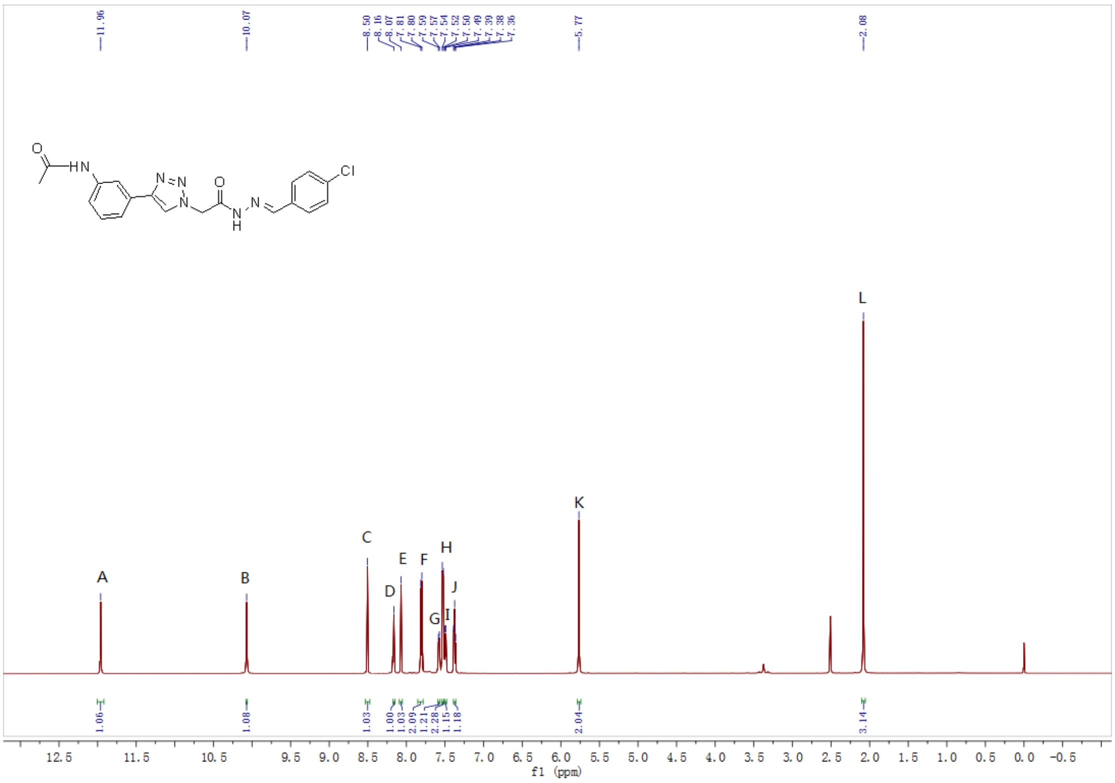

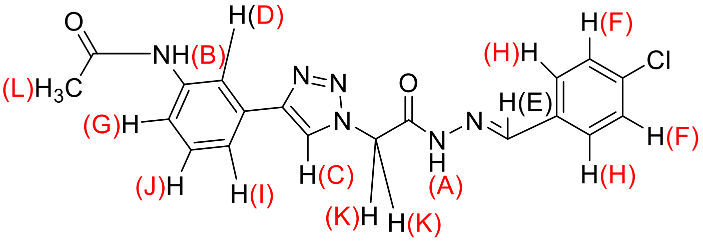

Assign.	Shift(ppm)
A	11.96
B	10.07
C	8.50
D	8.16
E	8.07
F	7.81
G	7.58
H	7.53
I	7.49

J	7.38
K	5.77
L	2.08

J(F)=8.5Hz. *J*(G)=7.4Hz. *J*(H)=8.5Hz. *J*(I)=7.4Hz. *J*(J)=7.9Hz.

Compounds 10a to 10k detailed ^{1}H-NMR spectrum attribution (Take 10a as an example）

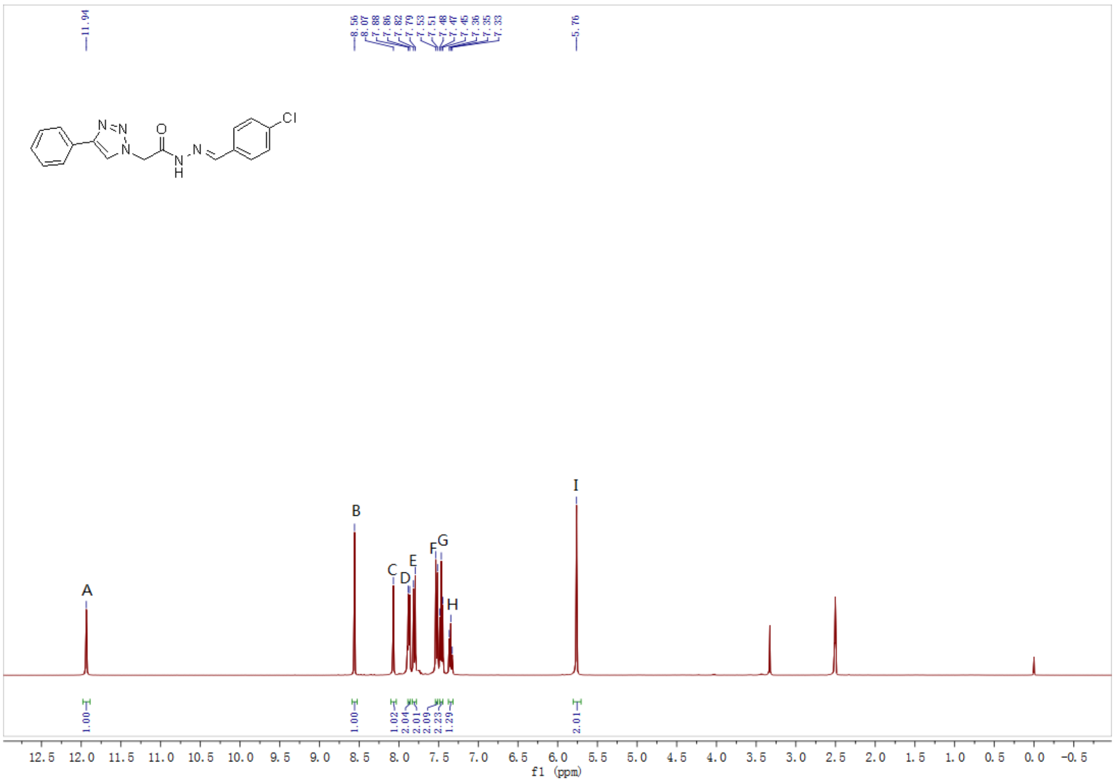

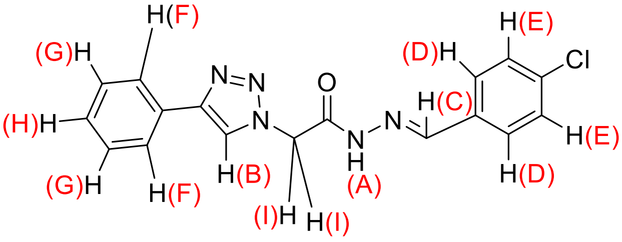

Assign.	Shift(ppm)
A	11.94
B	8.56
C	8.07

D	7.87
E	7.80
F	7.52
G	7.47
H	7.35
I	5.76

J(D)=7.1Hz. *J*(E)=8.5Hz. *J*(F)=8.5Hz. *J*(G)=7.7Hz. *J*(H)=7.4Hz.

Compounds 11a to 11k detailed ^{1}H-NMR spectrum attribution (Take 11a as an example）

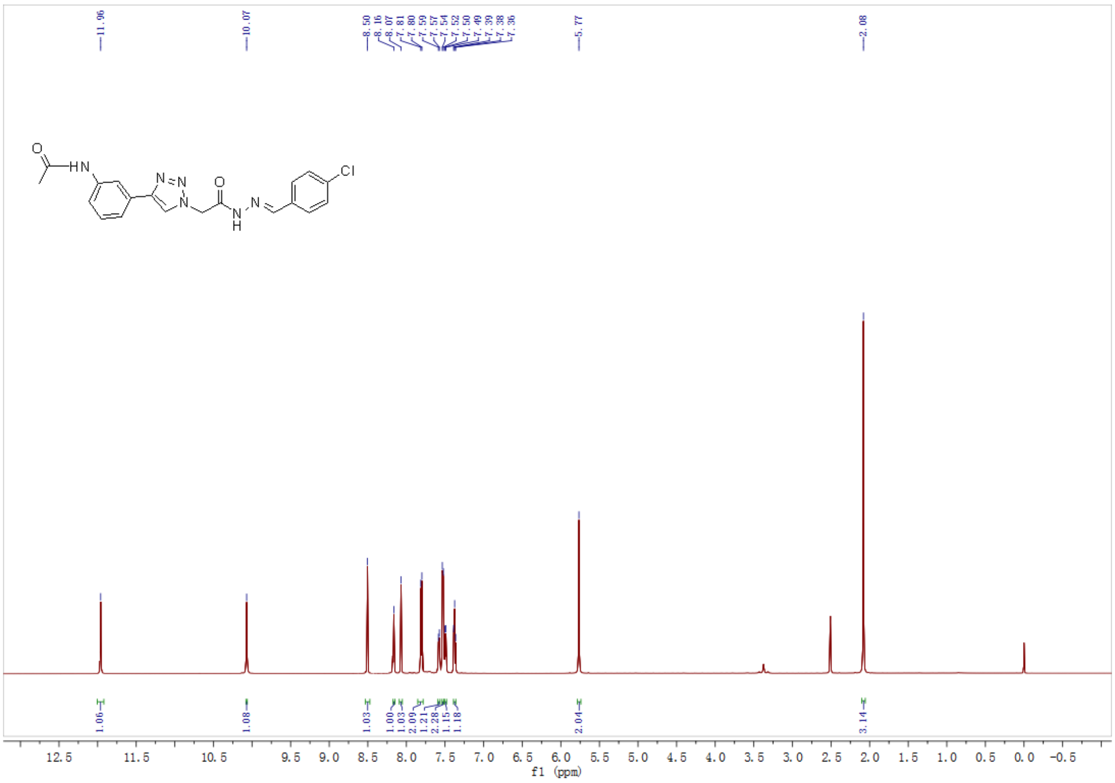

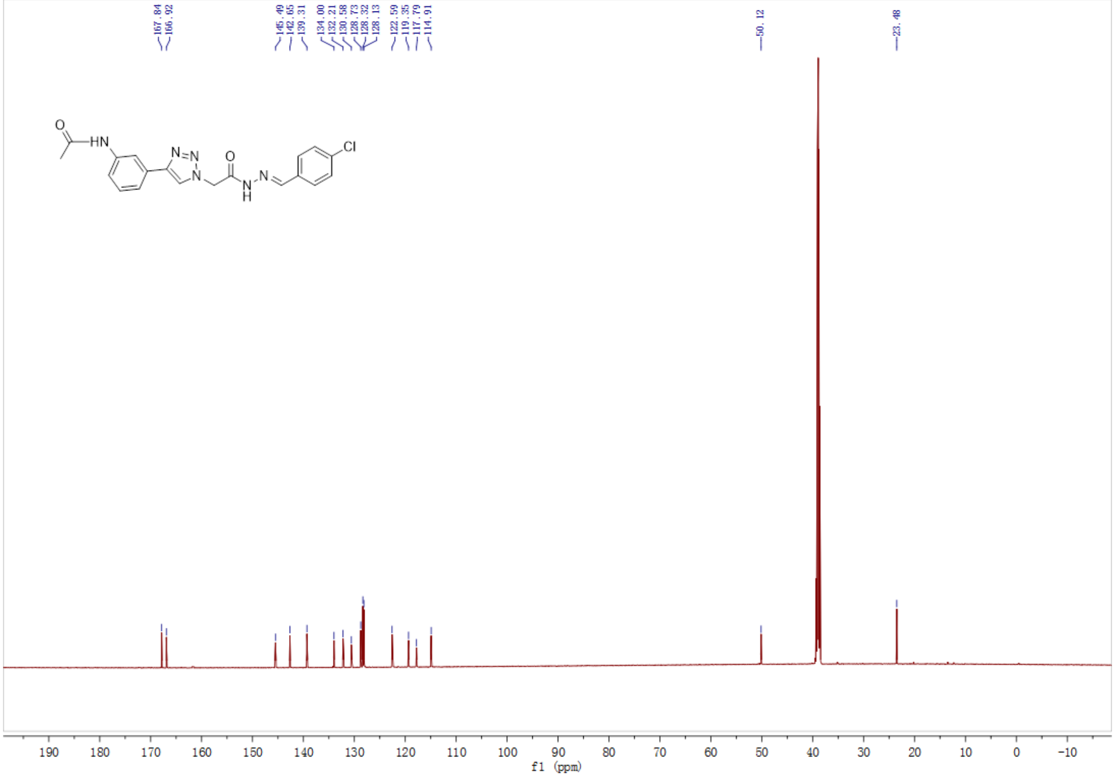

^{1}H-NMR spectrum of compound 10a

^{13}C-NMR spectrum of compound 10a

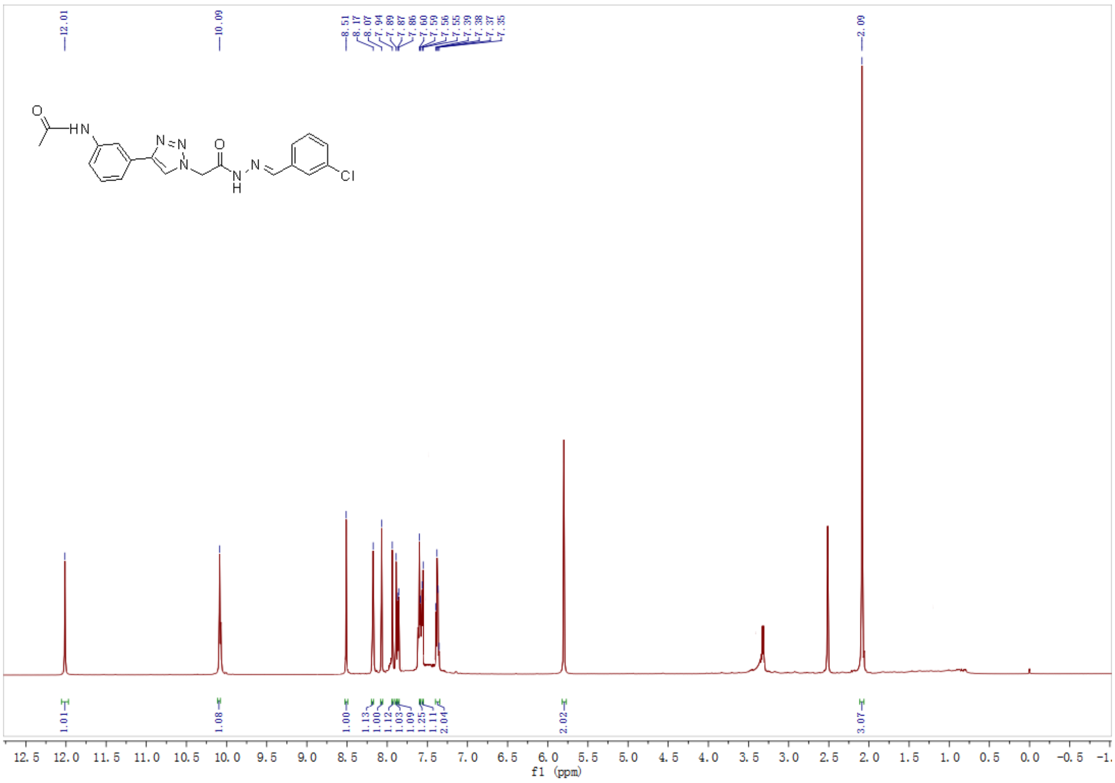

^{1}H-NMR spectrum of compound 10b

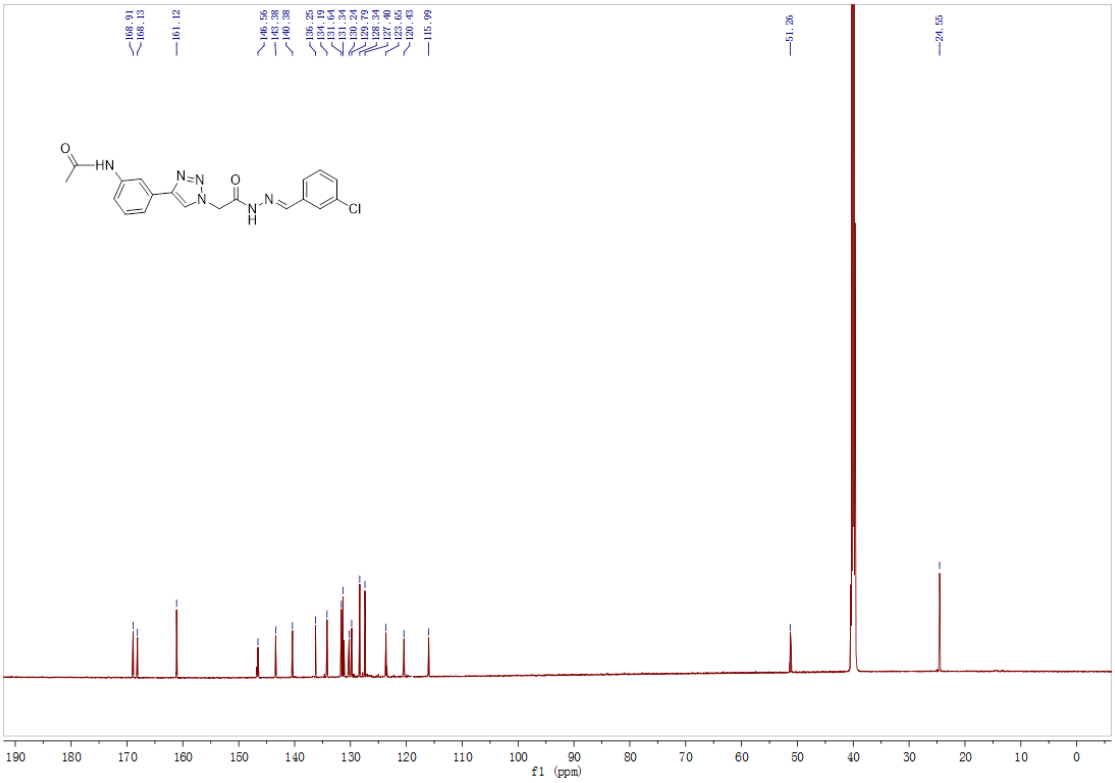

^{13}C-NMR spectrum of compound 10b

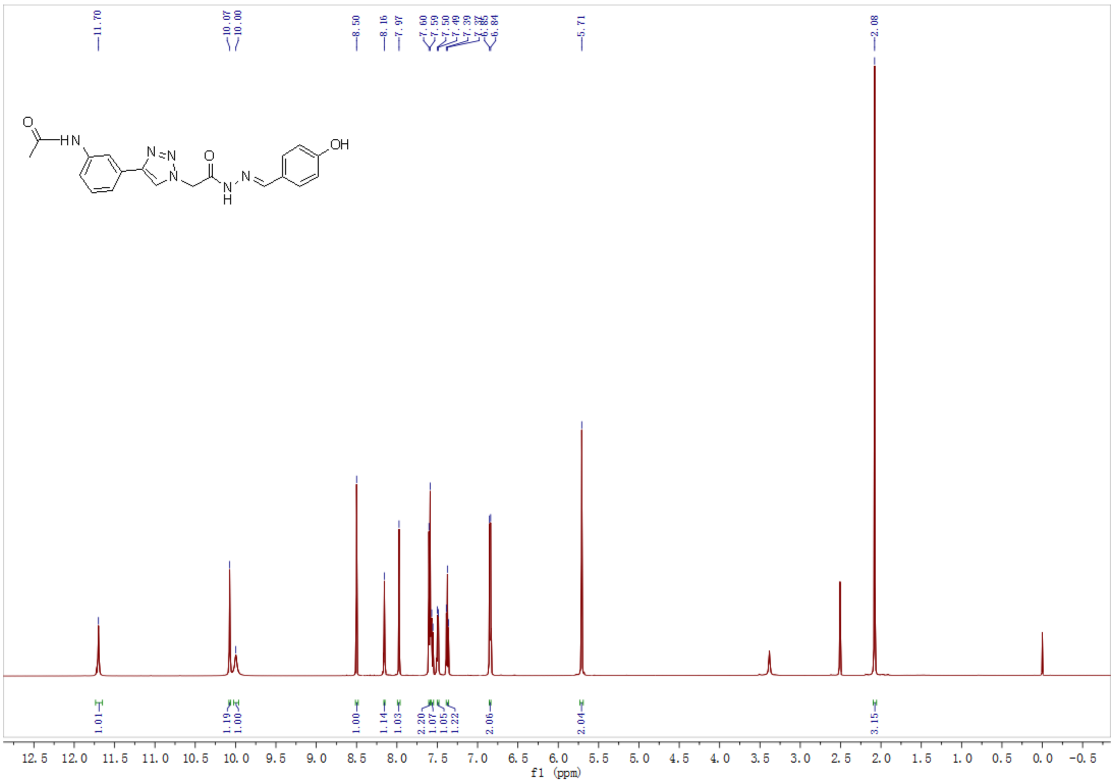

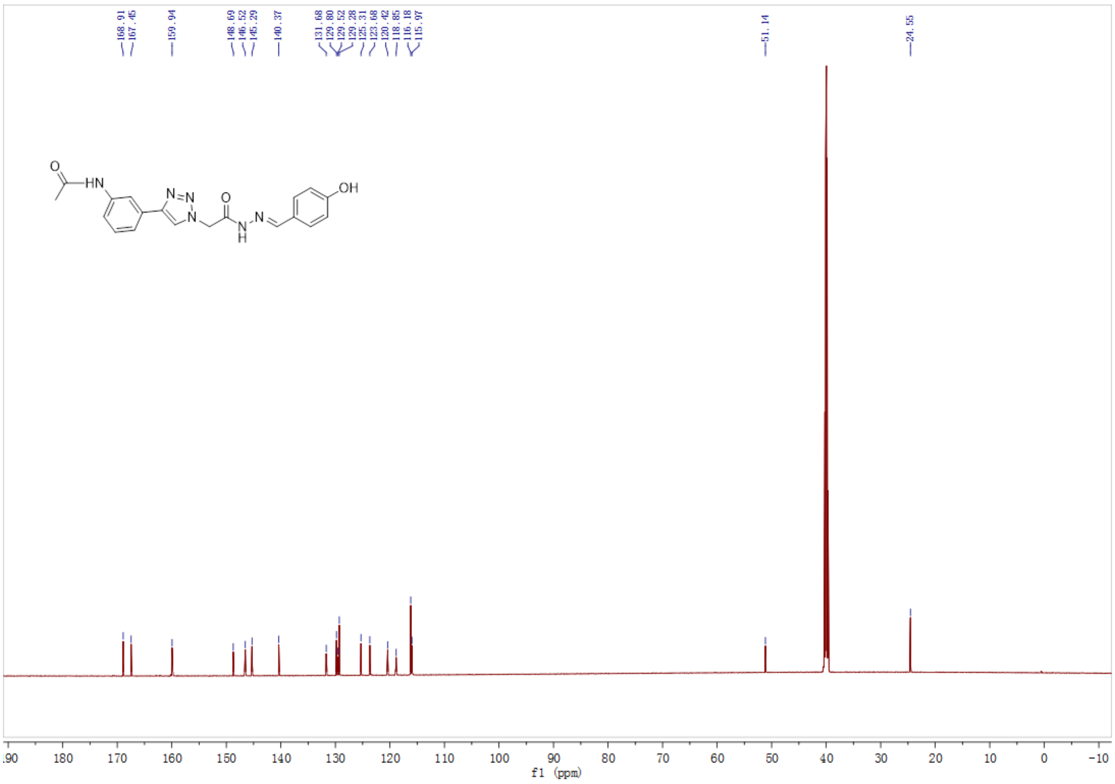

^{1}H-NMR spectrum of compound 10c

^{13}C-NMR spectrum of compound 10c

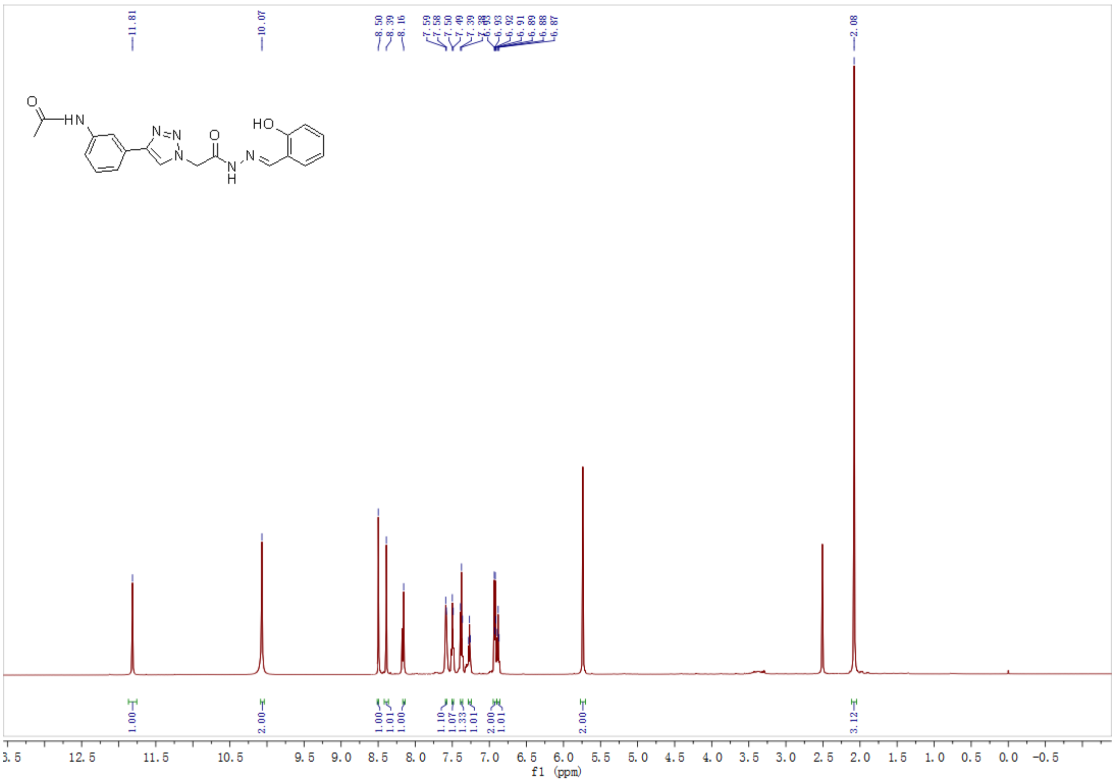

^{1}H-NMR spectrum of compound 10d

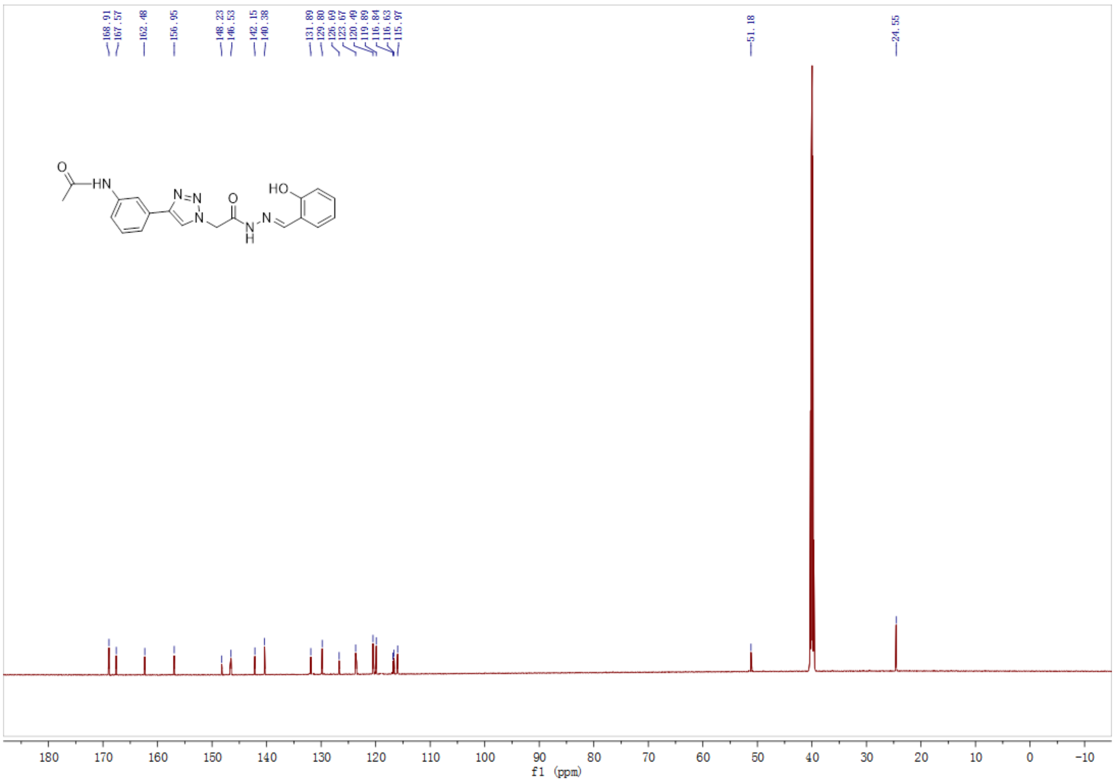

^{13}C-NMR spectrum of compound 10d

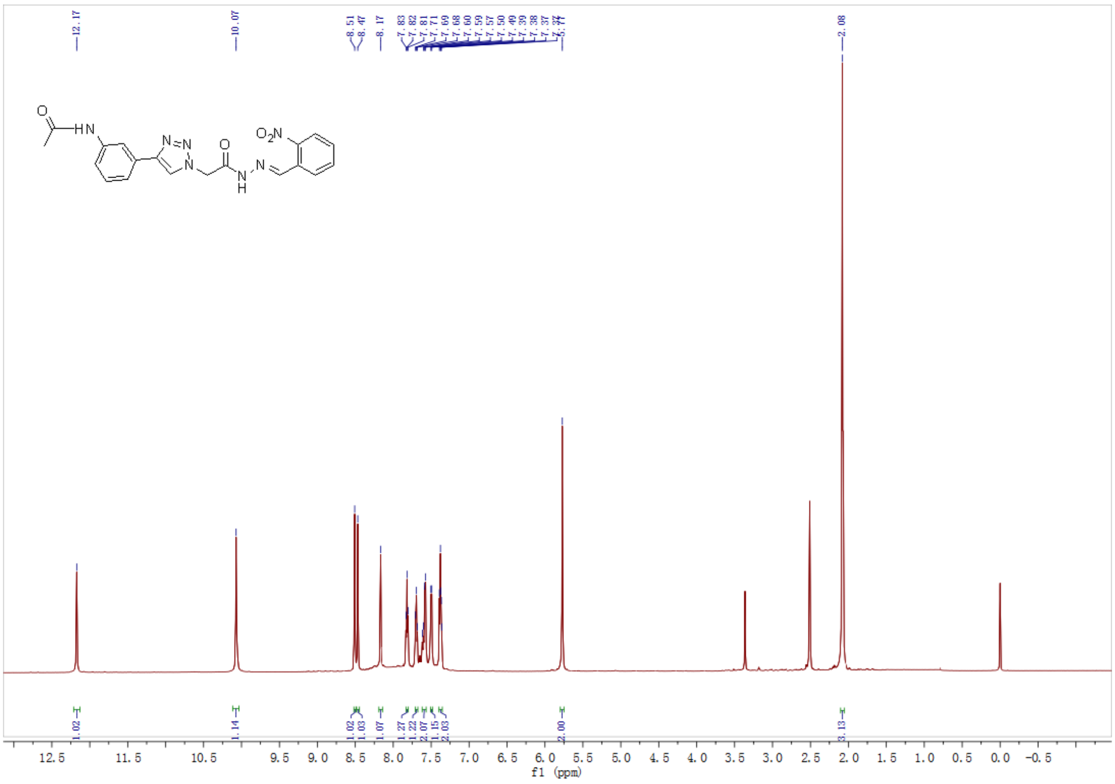

^{1}H-NMR spectrum of compound 10e

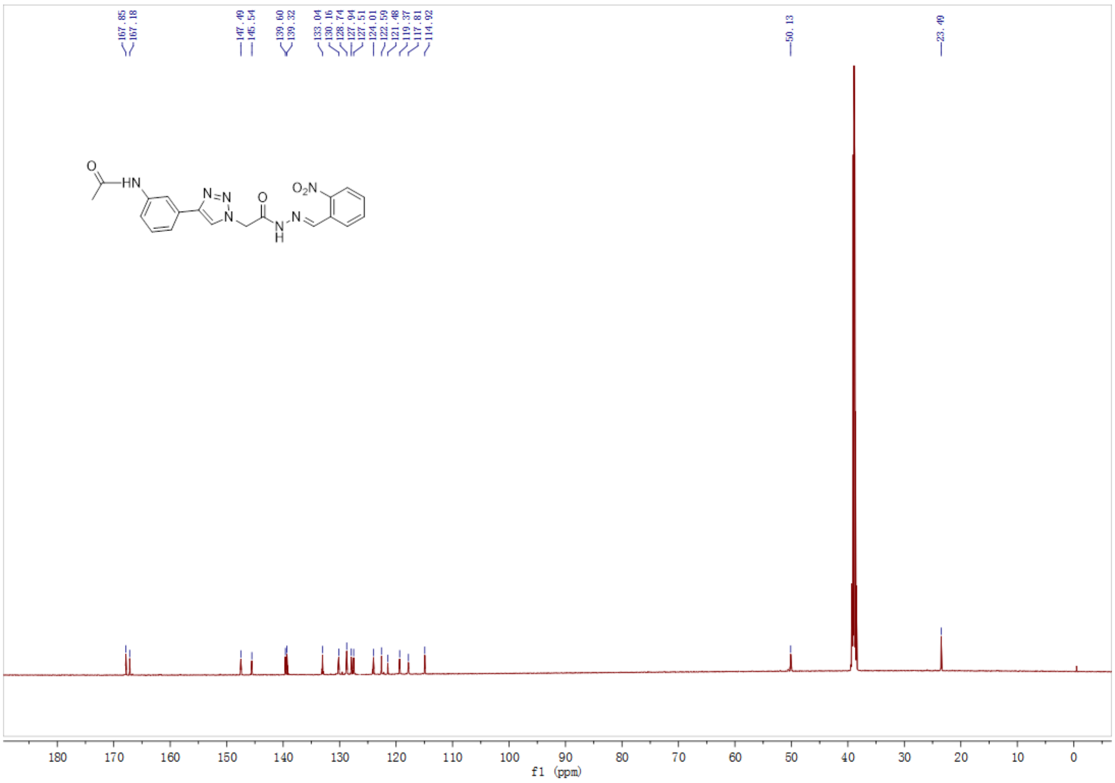

^{13}C-NMR spectrum of compound 10e

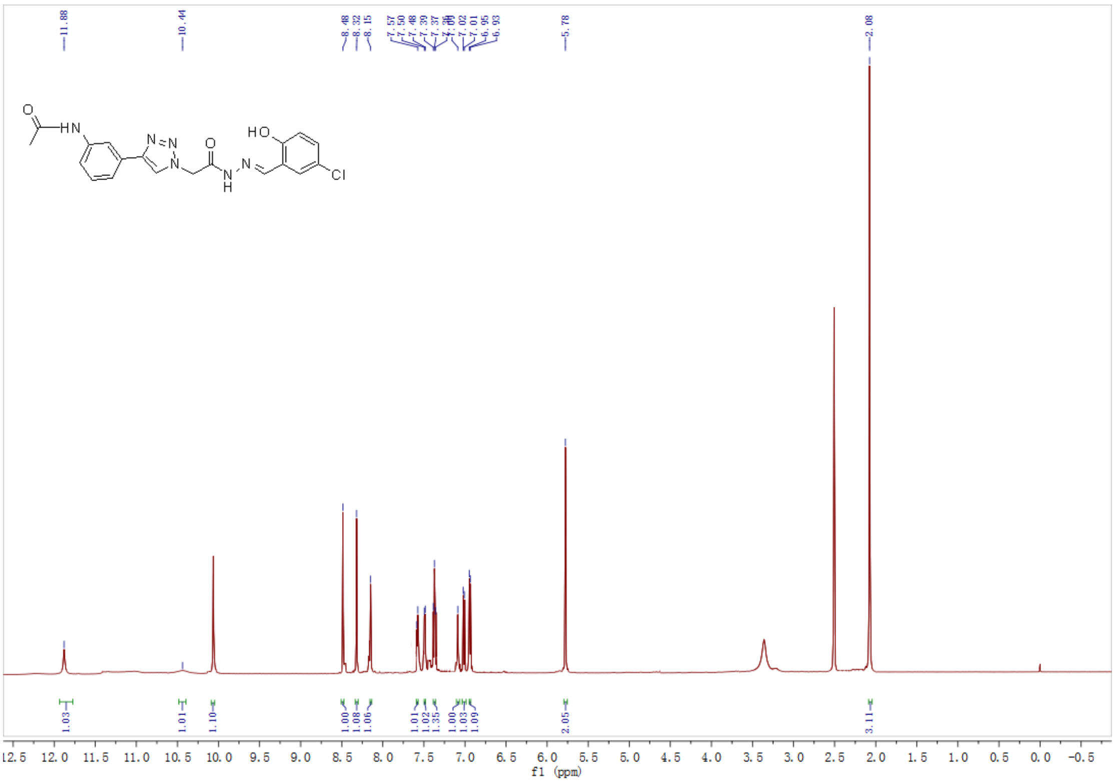

^{1}H-NMR spectrum of compound 10f

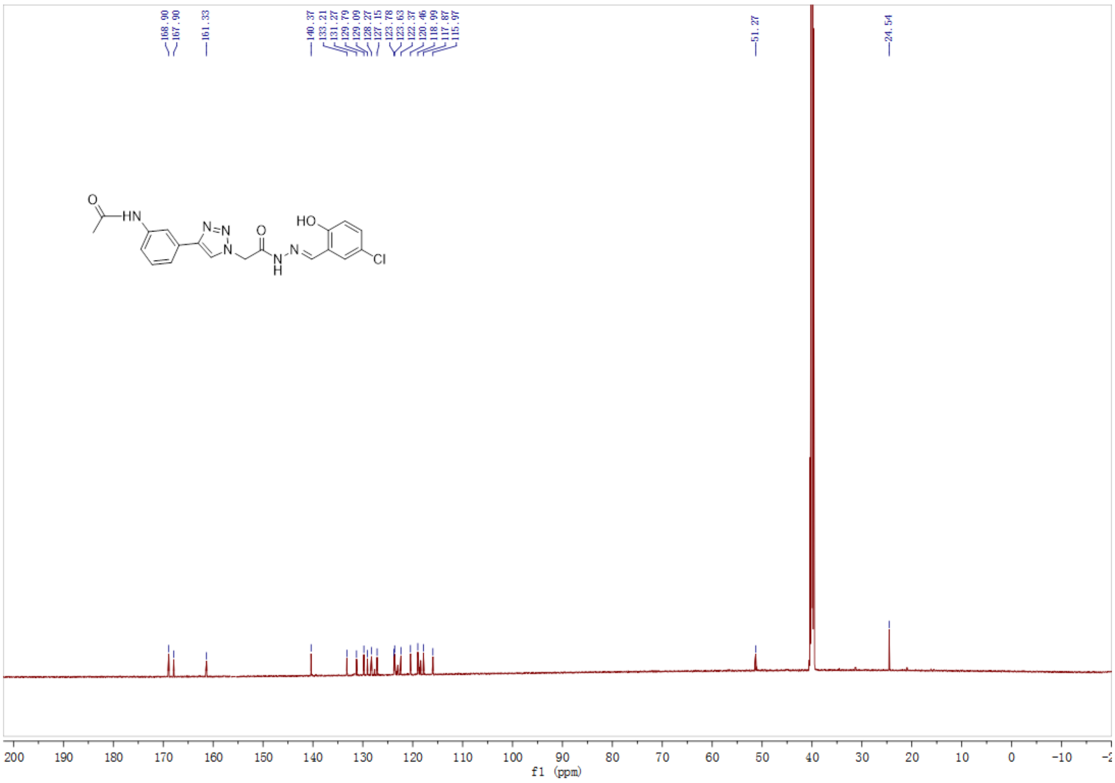

^{13}C-NMR spectrum of compound 10f

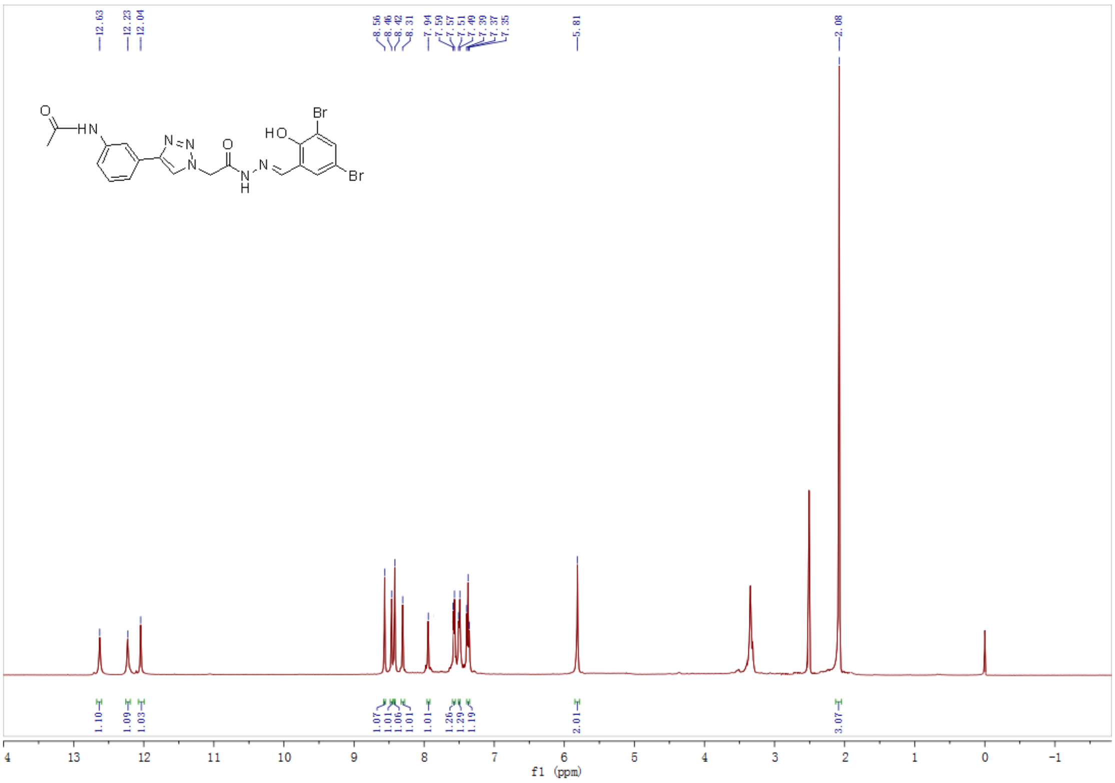

^{1}H-NMR spectrum of compound 10g

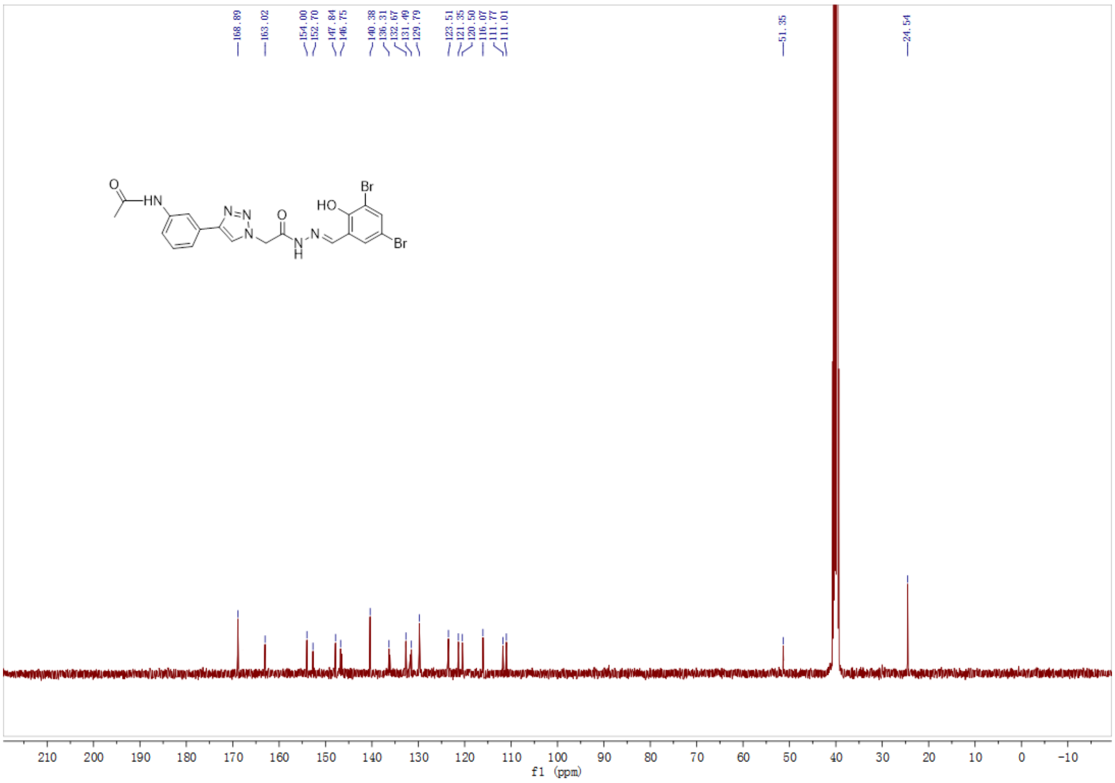

^{13}C-NMR spectrum of compound 10g

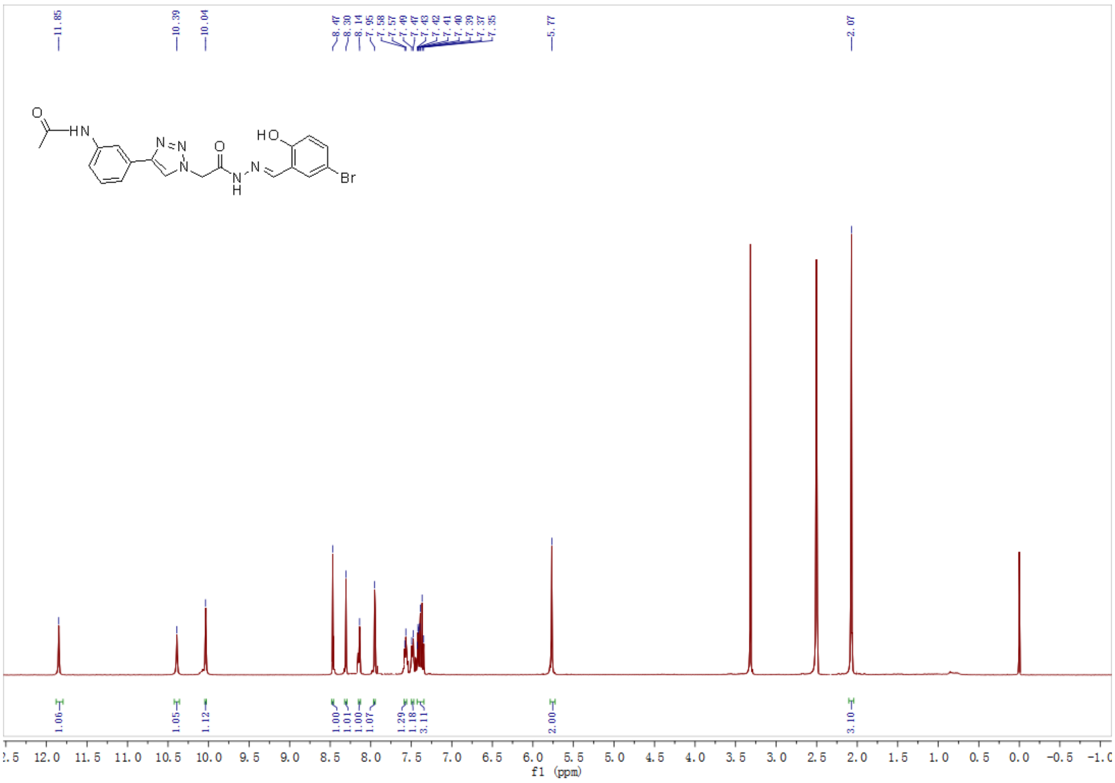

^{1}H-NMR spectrum of compound 10h

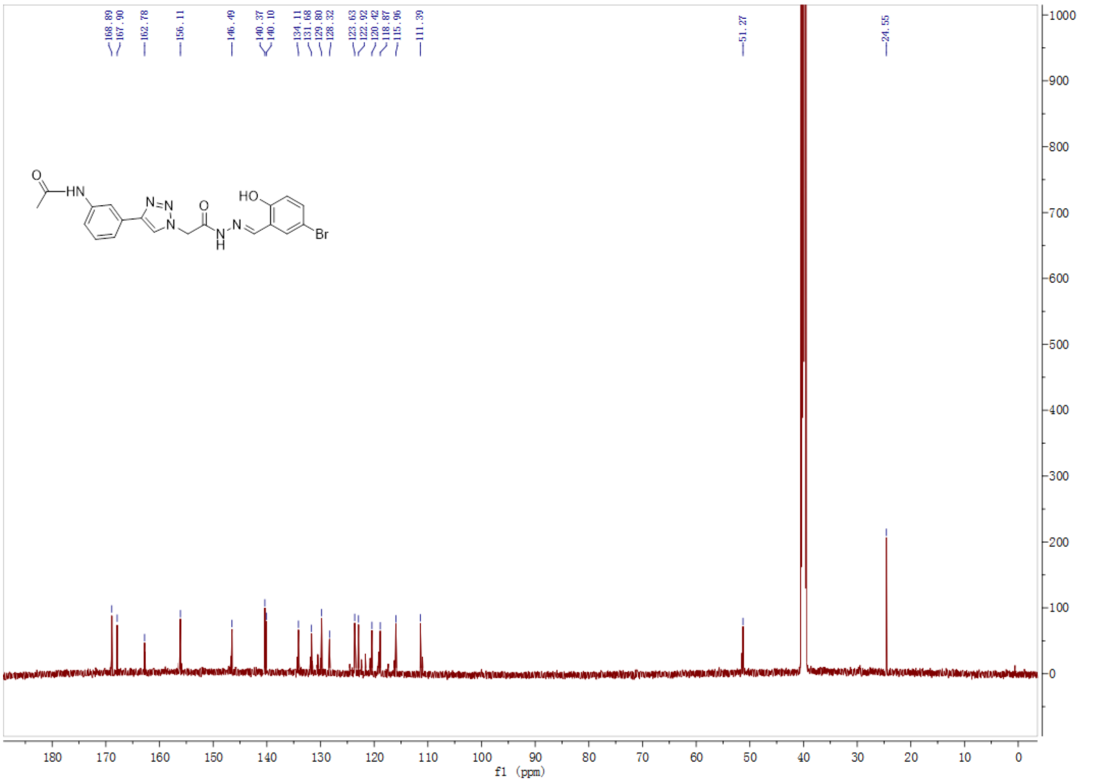

^{13}C-NMR spectrum of compound 10h

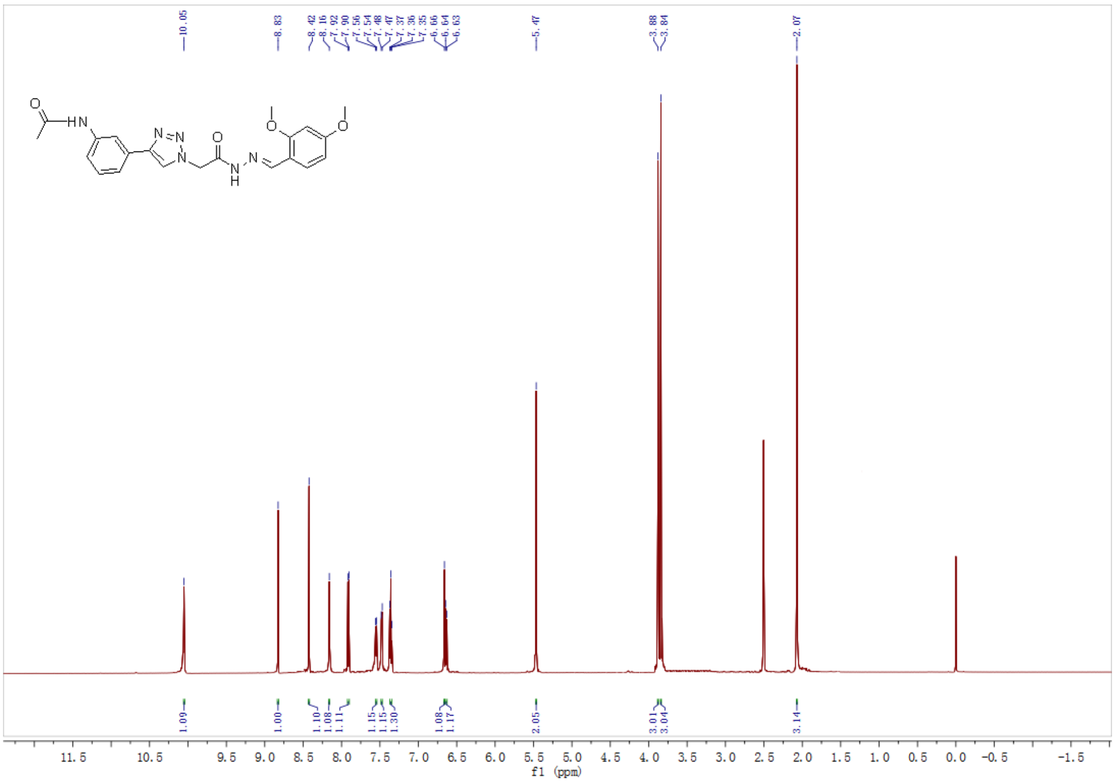

^{1}H-NMR spectrum of compound 10i

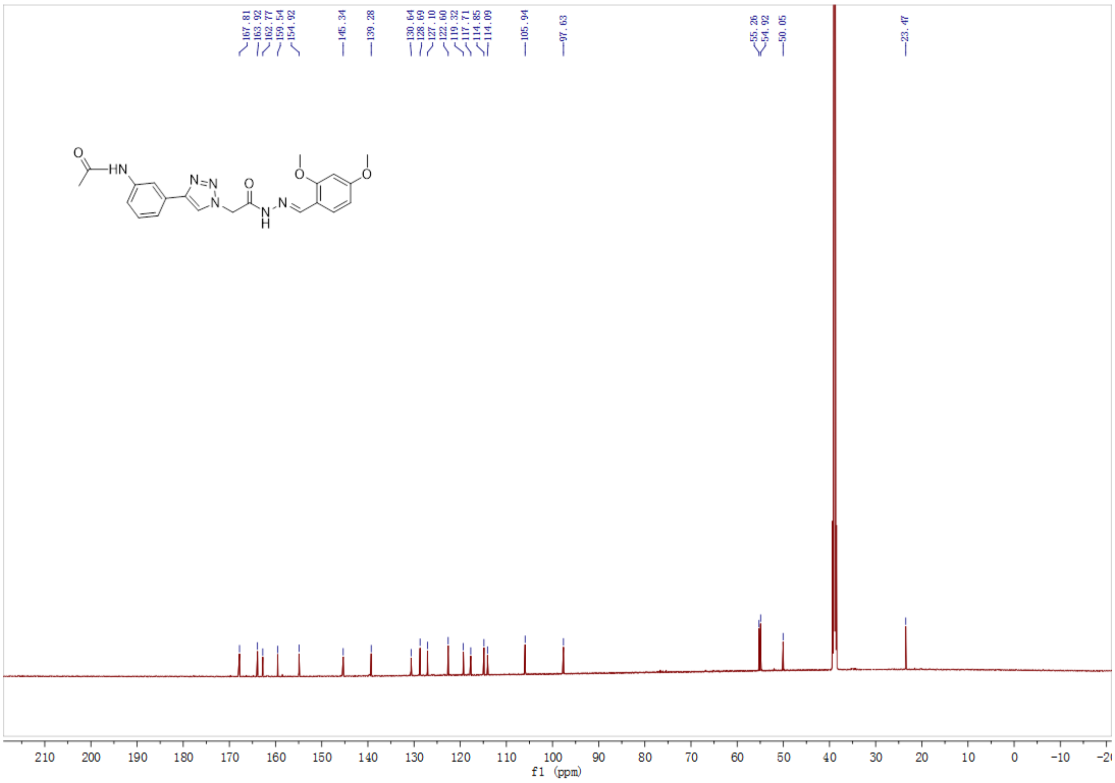

^{13}C-NMR spectrum of compound 10i

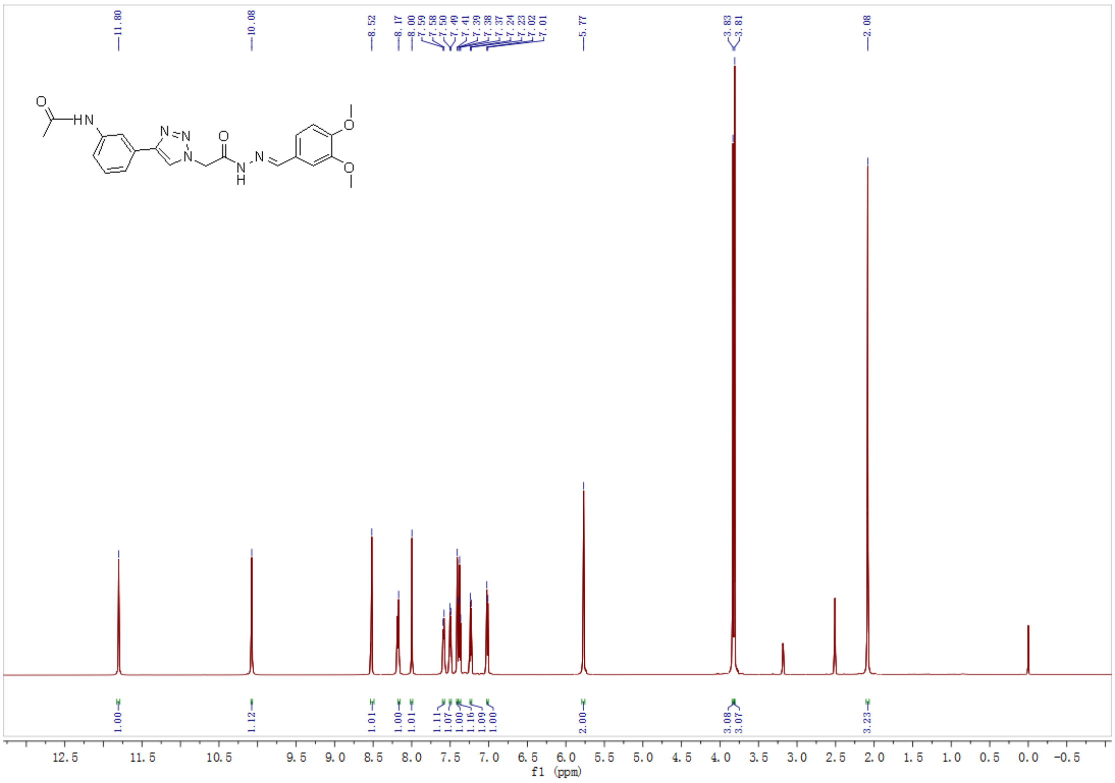

^{1}H-NMR spectrum of compound 10j

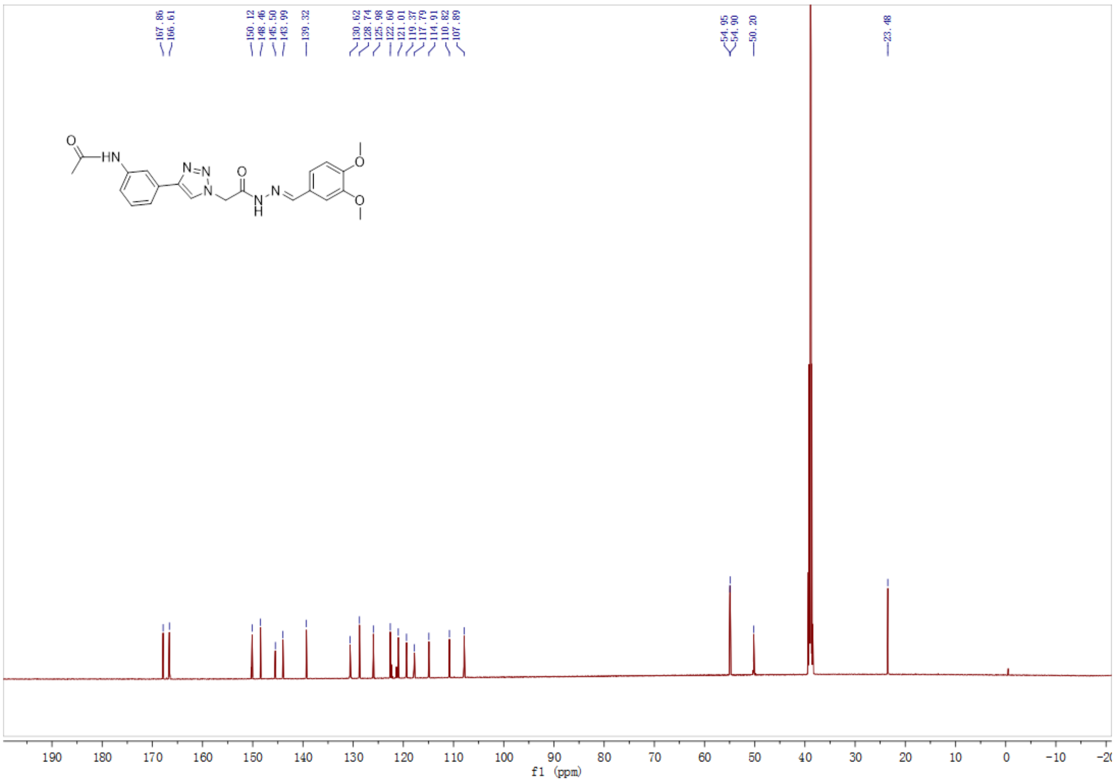

^{13}C-NMR spectrum of compound 10j

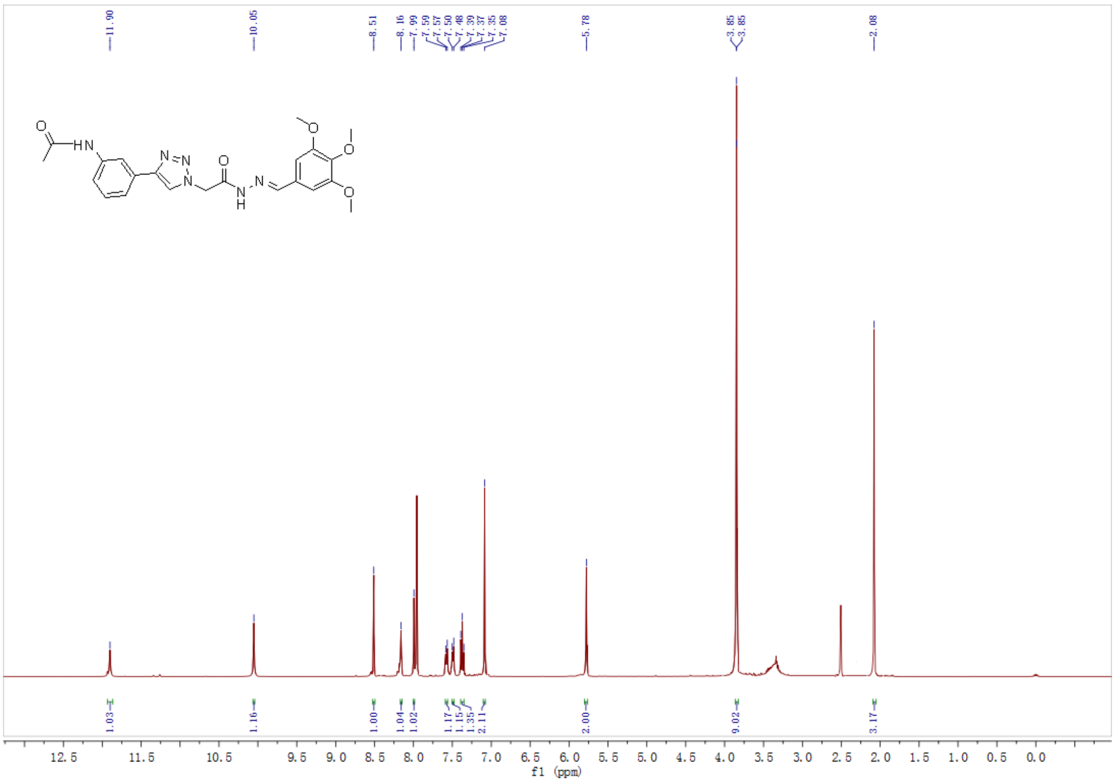

^{1}H-NMR spectrum of compound 10k

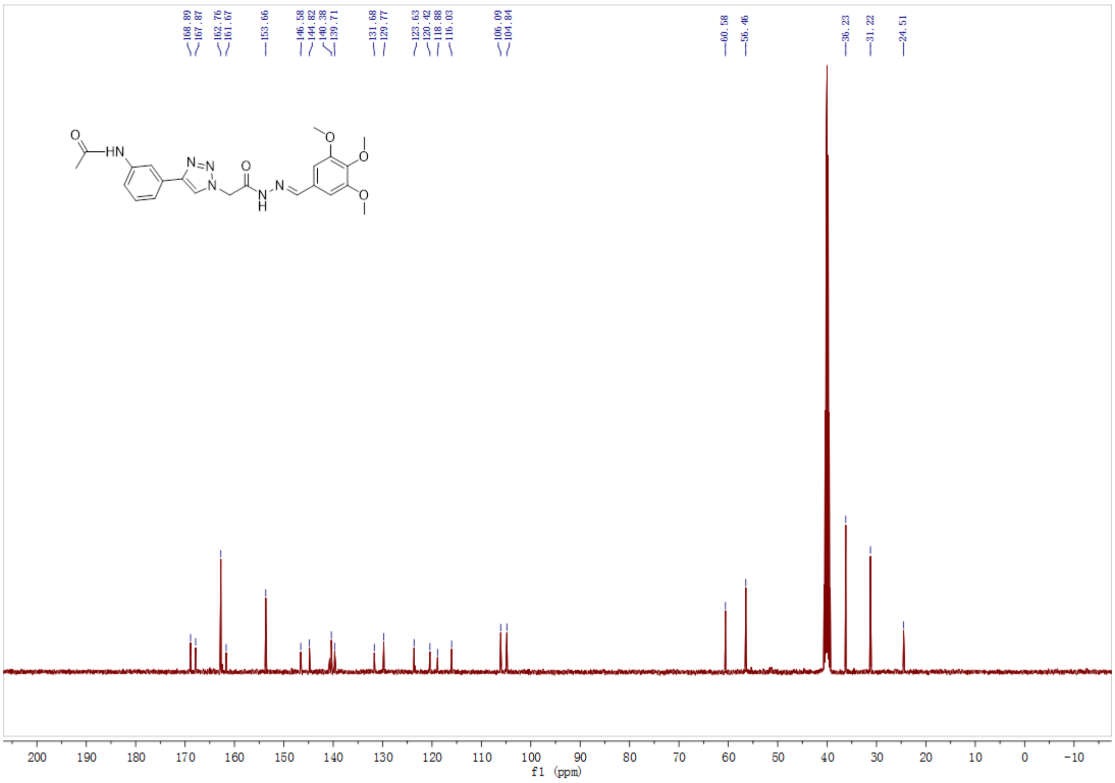

^{13}C-NMR spectrum of compound 10k

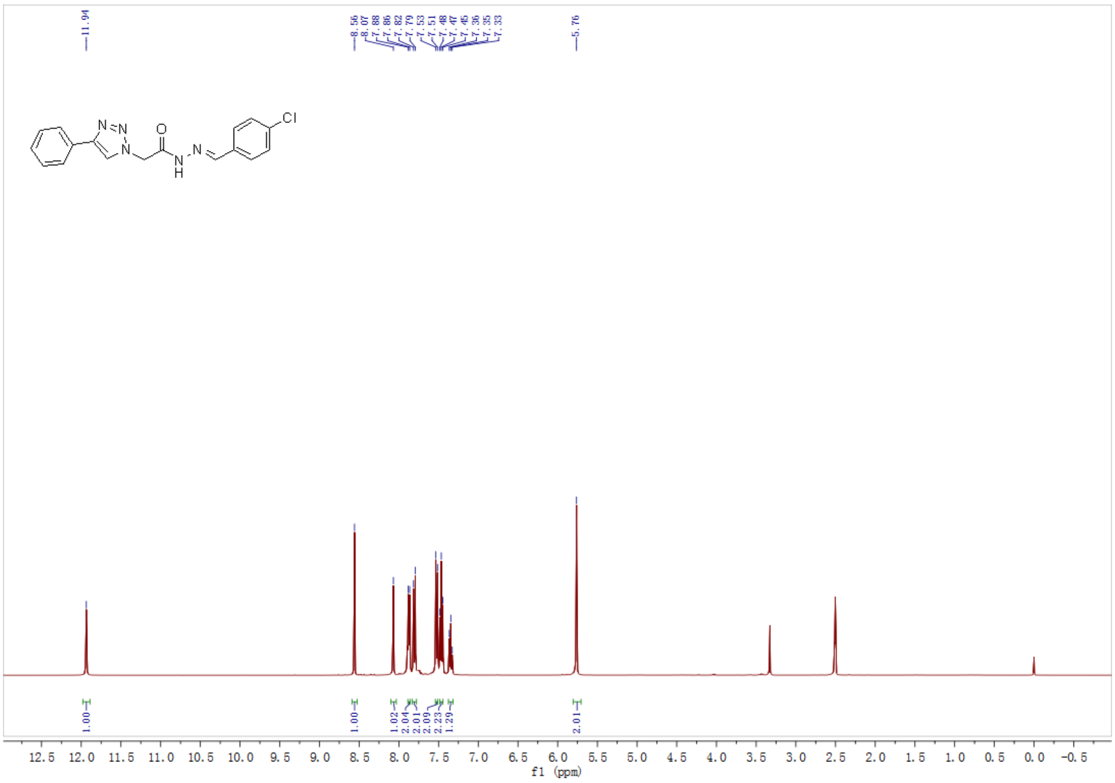

^{1}H-NMR spectrum of compound 11a

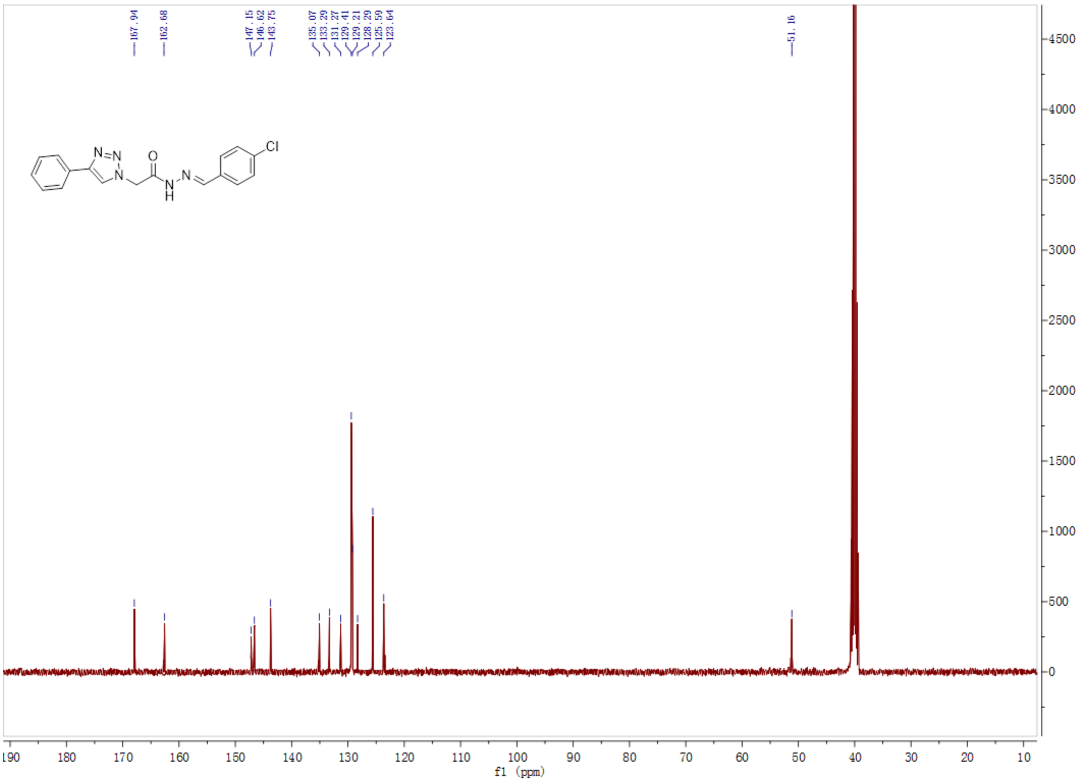

^{13}C-NMR spectrum of compound 11a

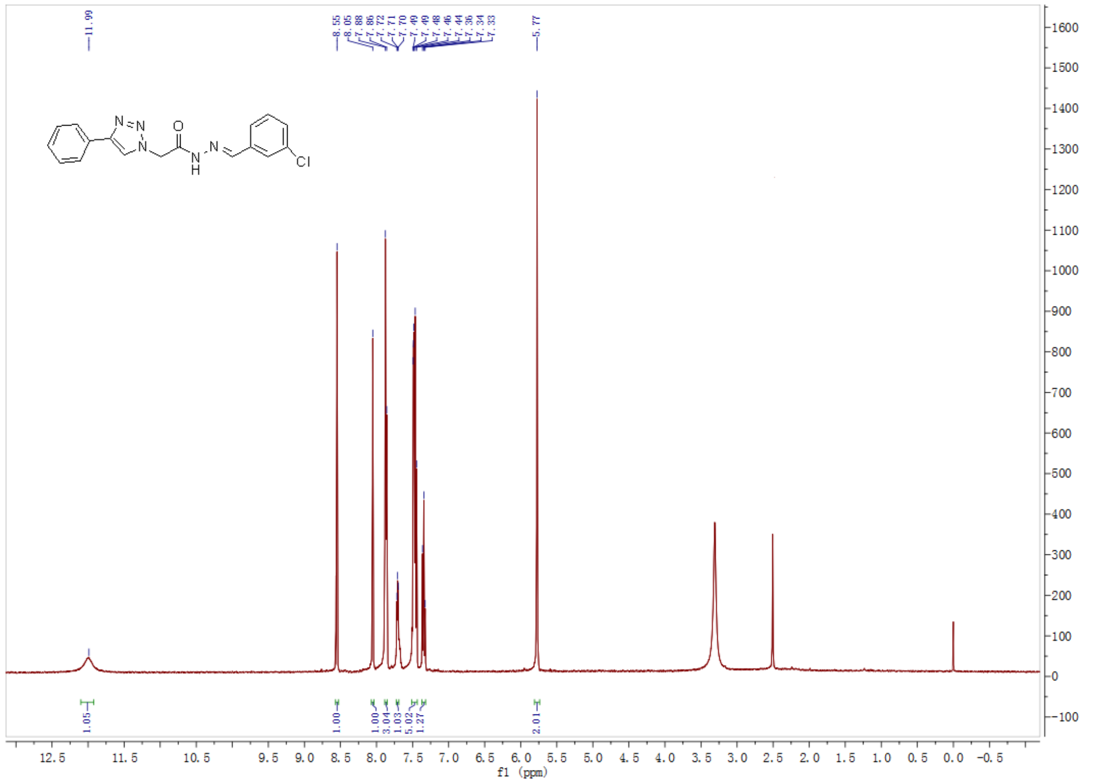

^{1}H-NMR spectrum of compound 11b

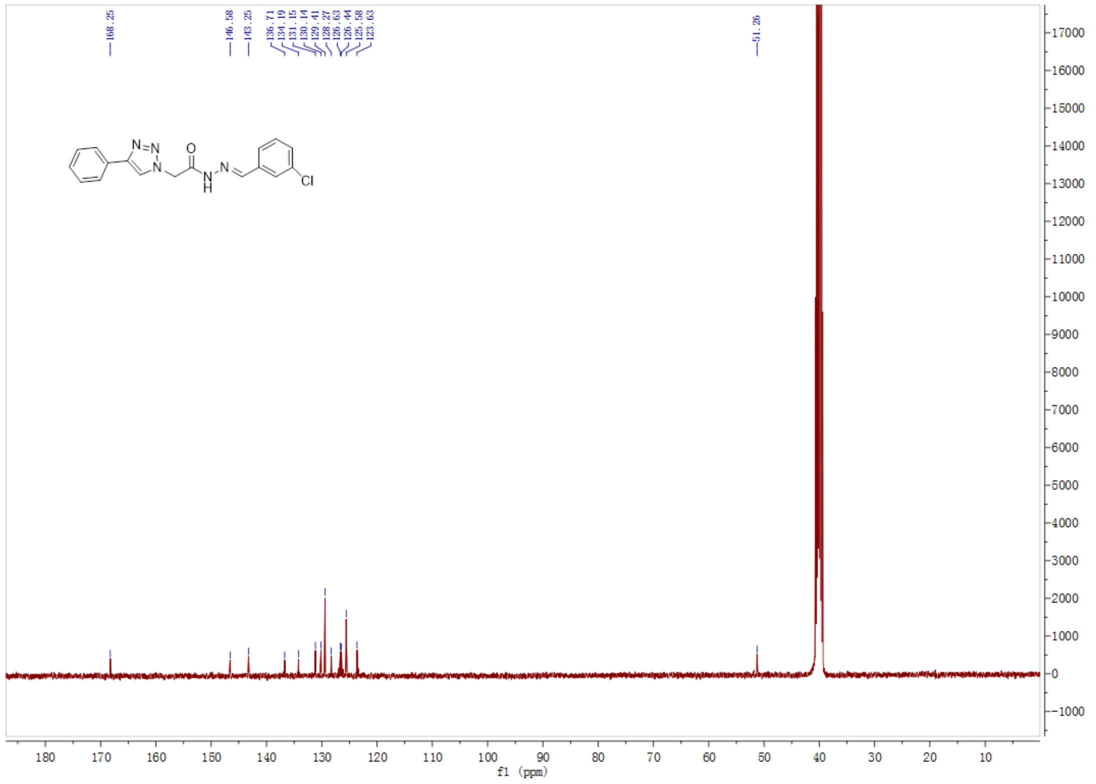

^{13}C-NMR spectrum of compound 11b

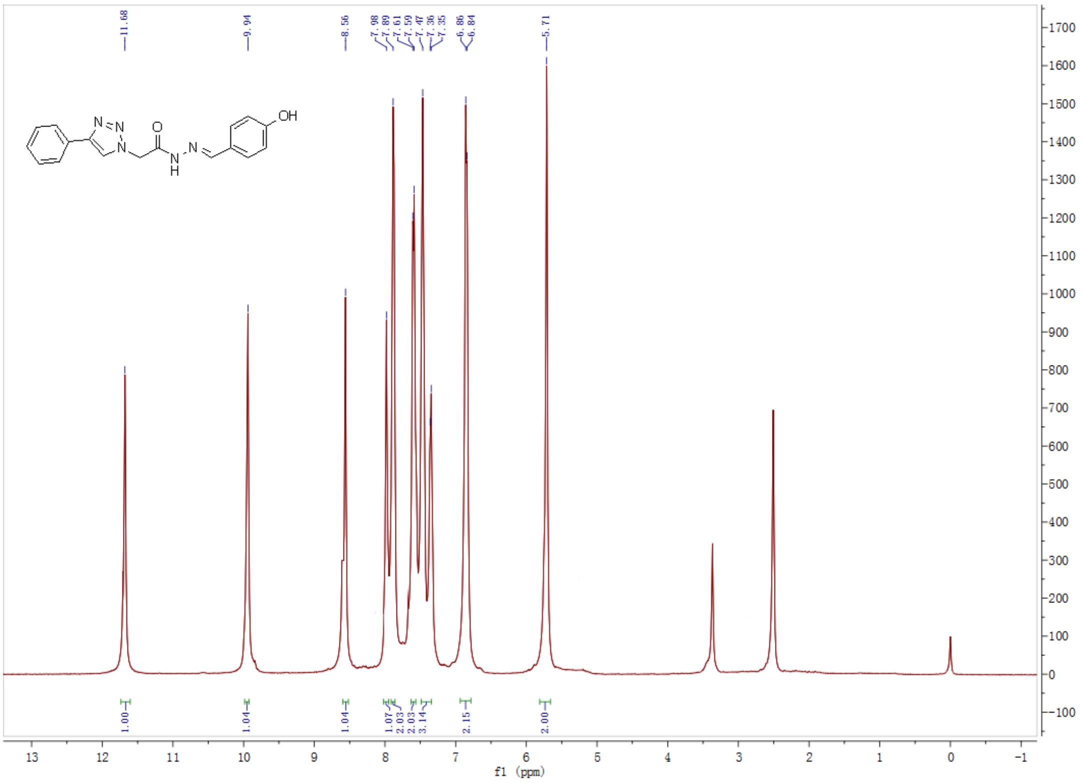

^{1}H-NMR spectrum of compound 11c

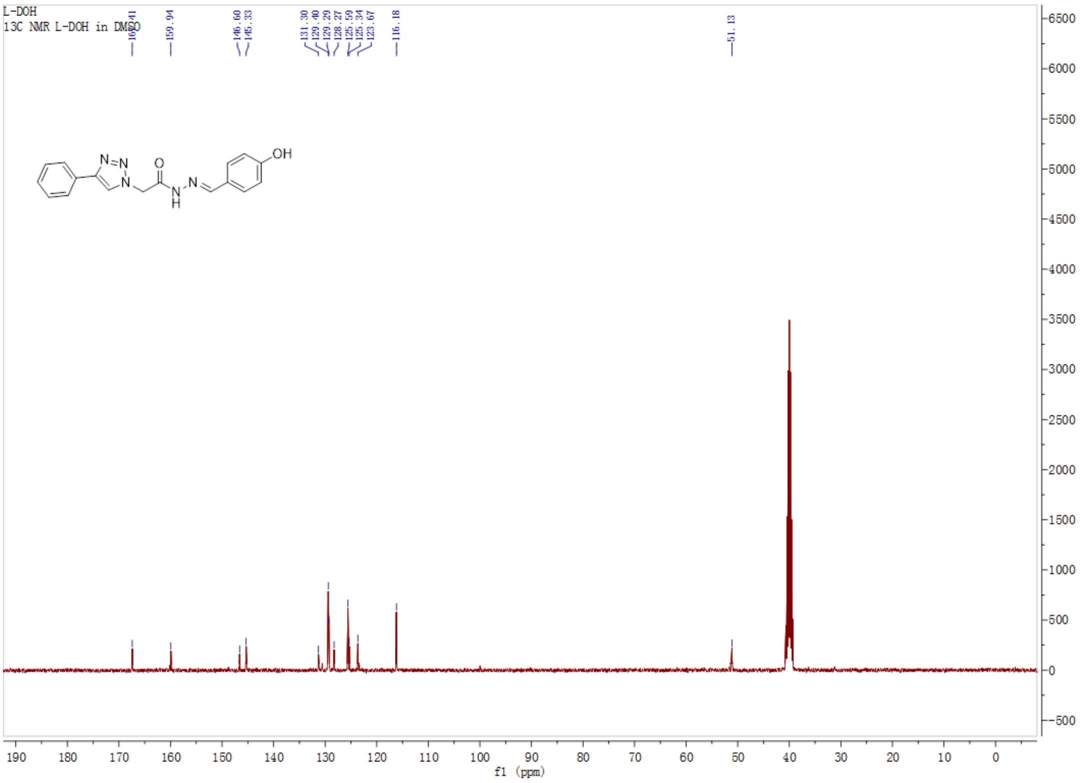

^{13}C-NMR spectrum of compound 11c

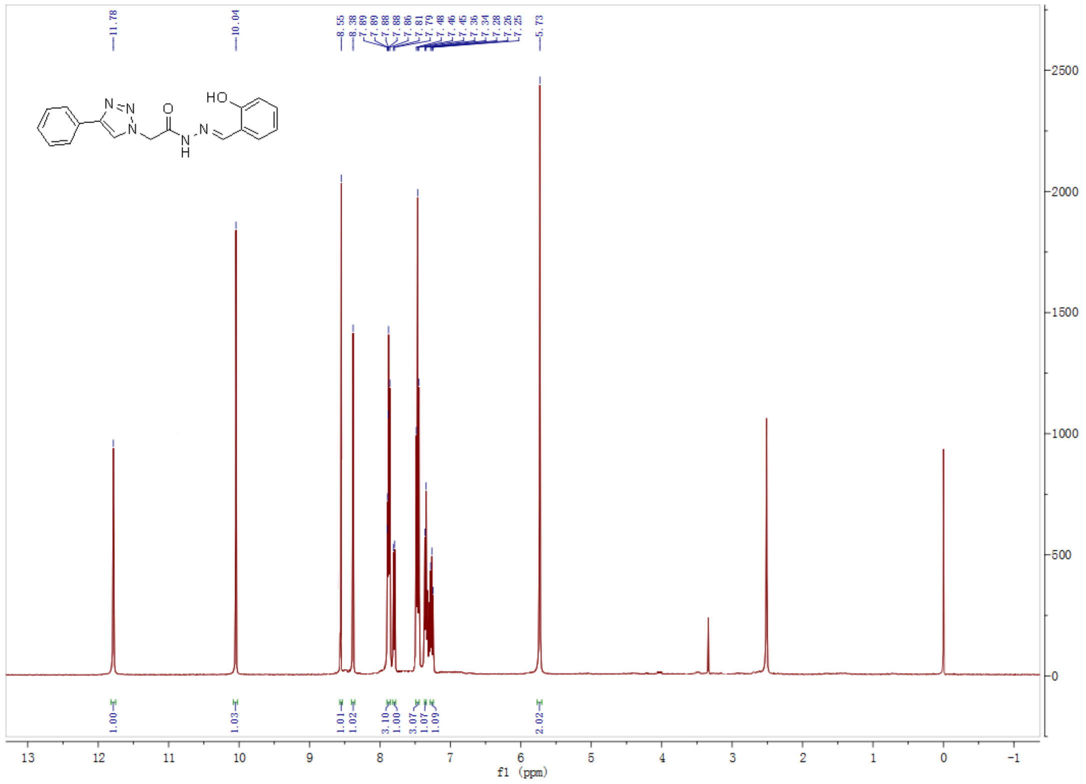

^{1}H-NMR spectrum of compound 11d

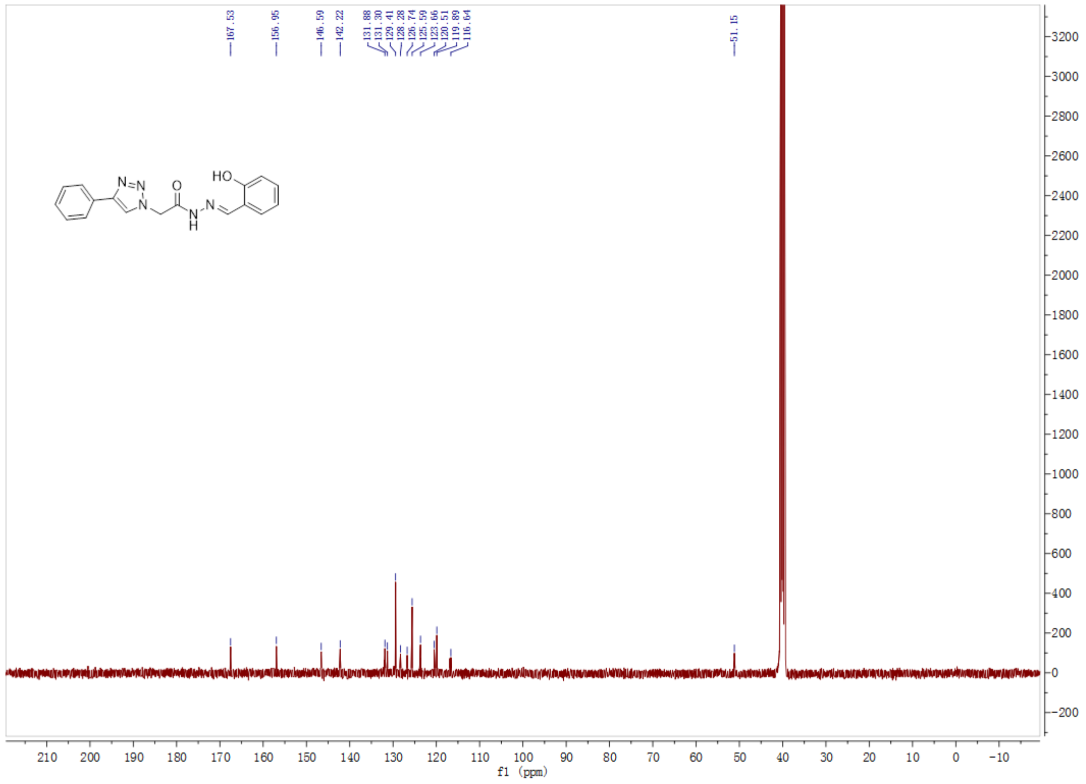

^{13}C-NMR spectrum of compound 11d

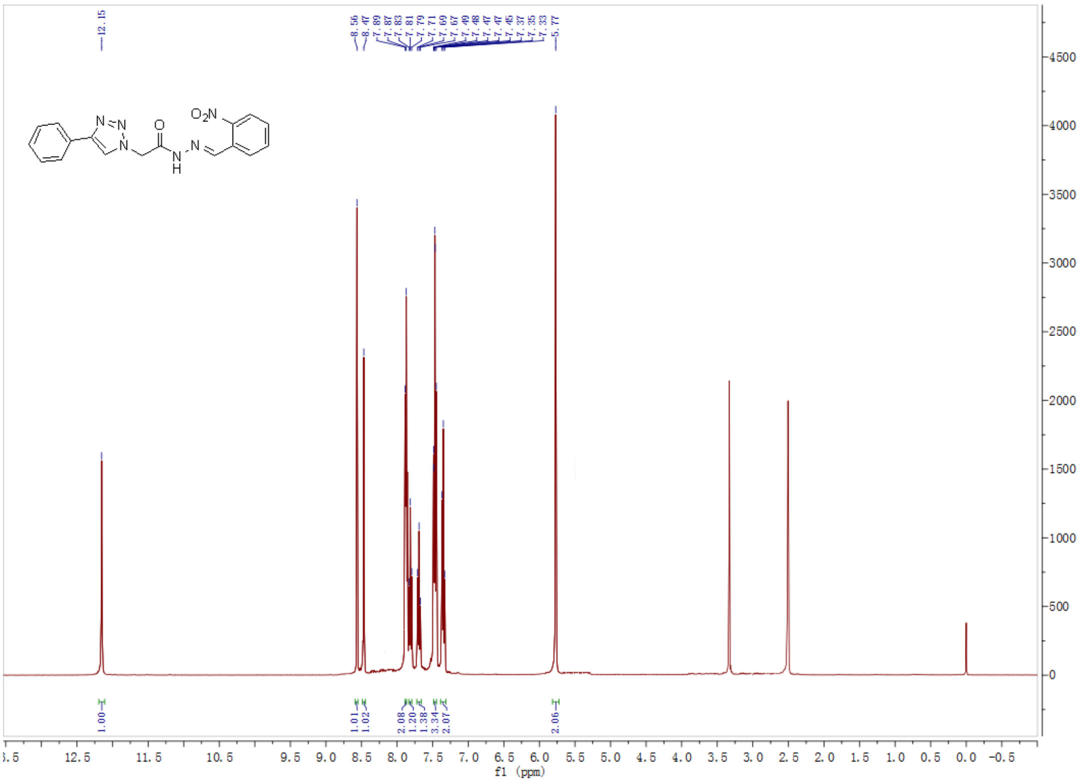

^{1}H-NMR spectrum of compound 11e

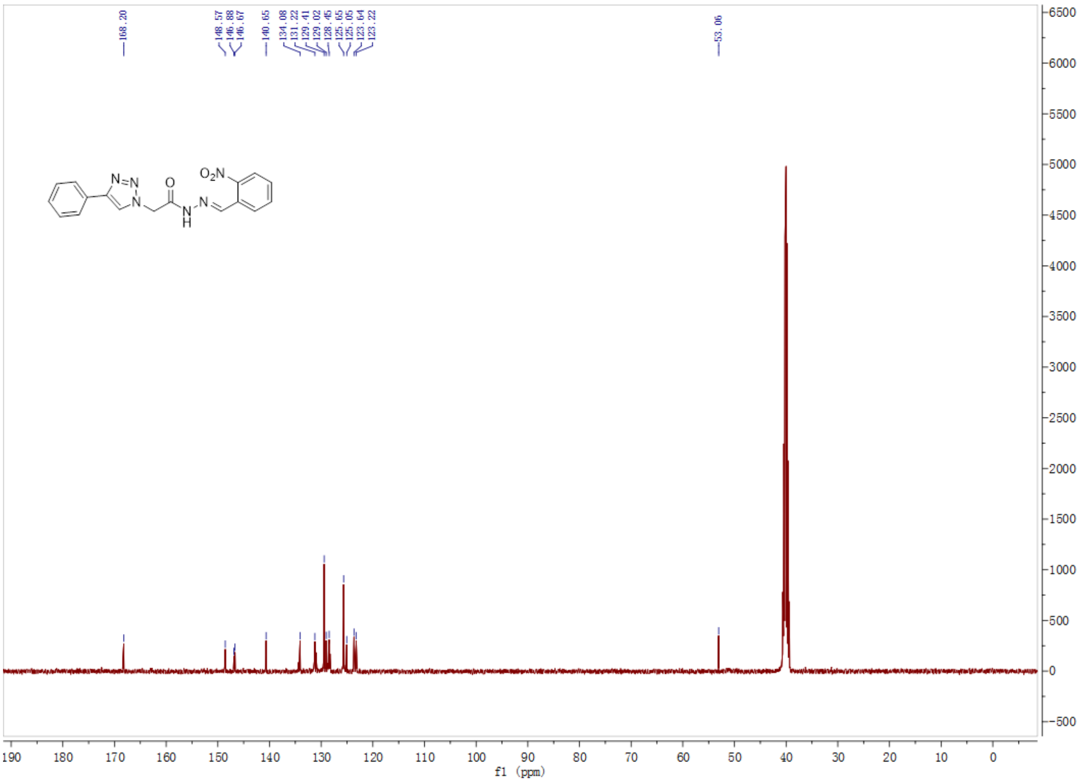

^{13}C-NMR spectrum of compound 11e

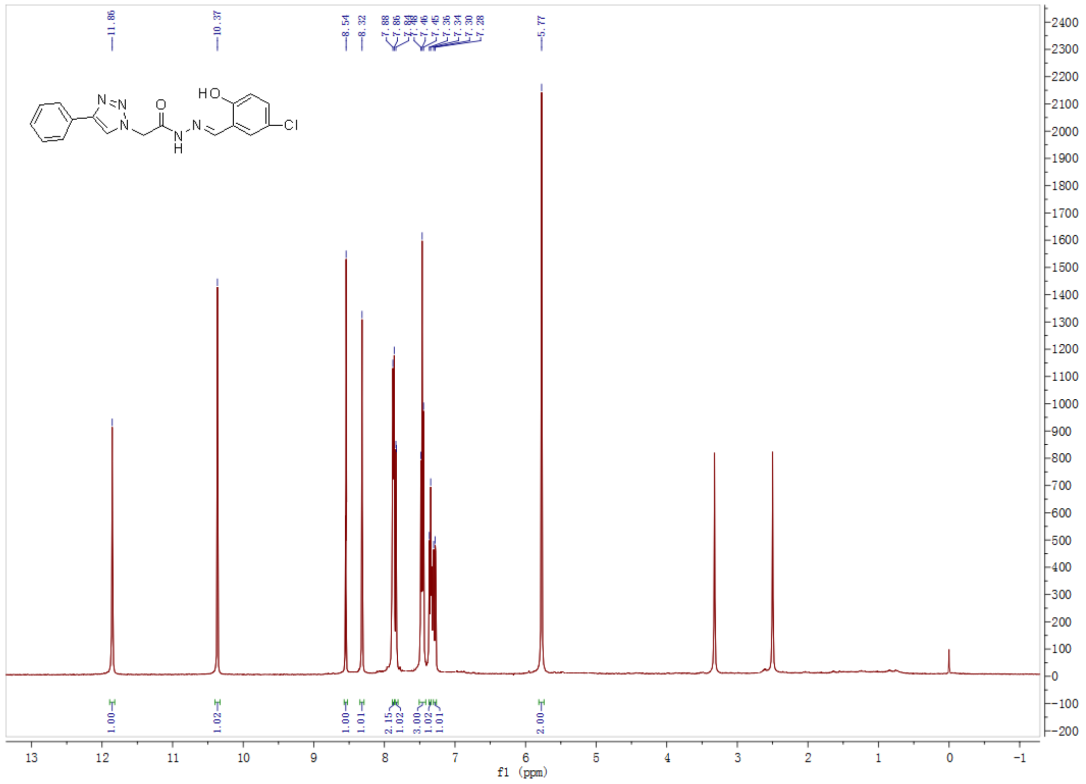

^{1}H-NMR spectrum of compound 11f

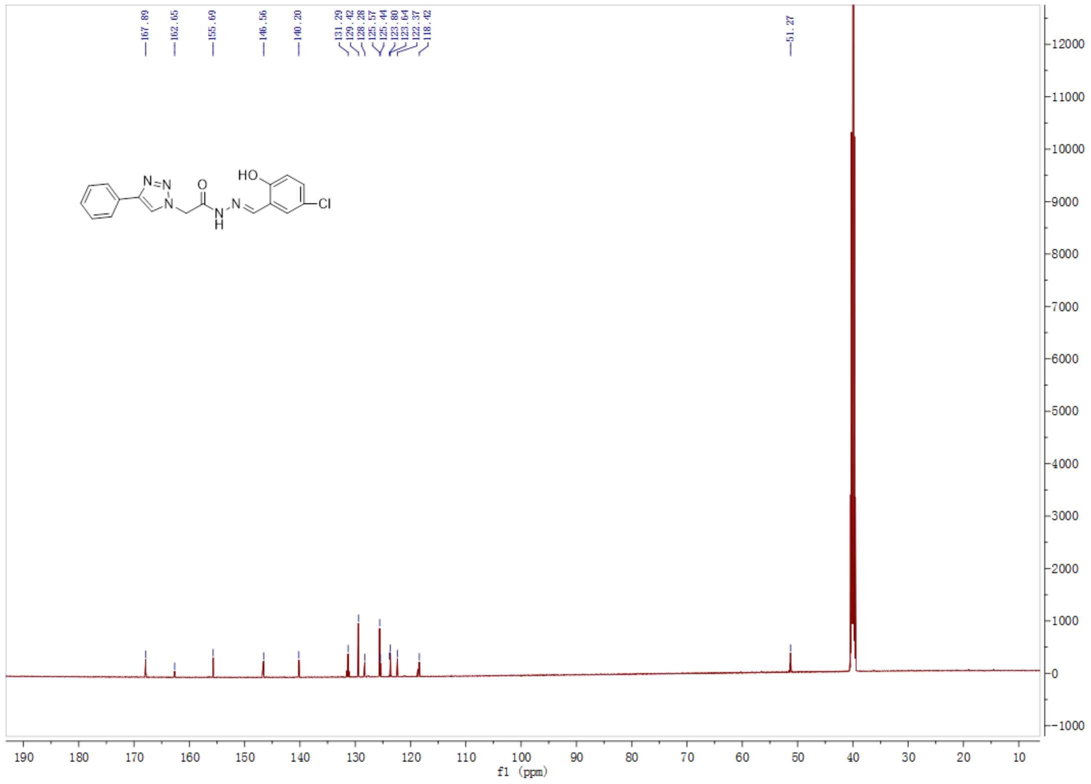

^{13}C-NMR spectrum of compound 11f

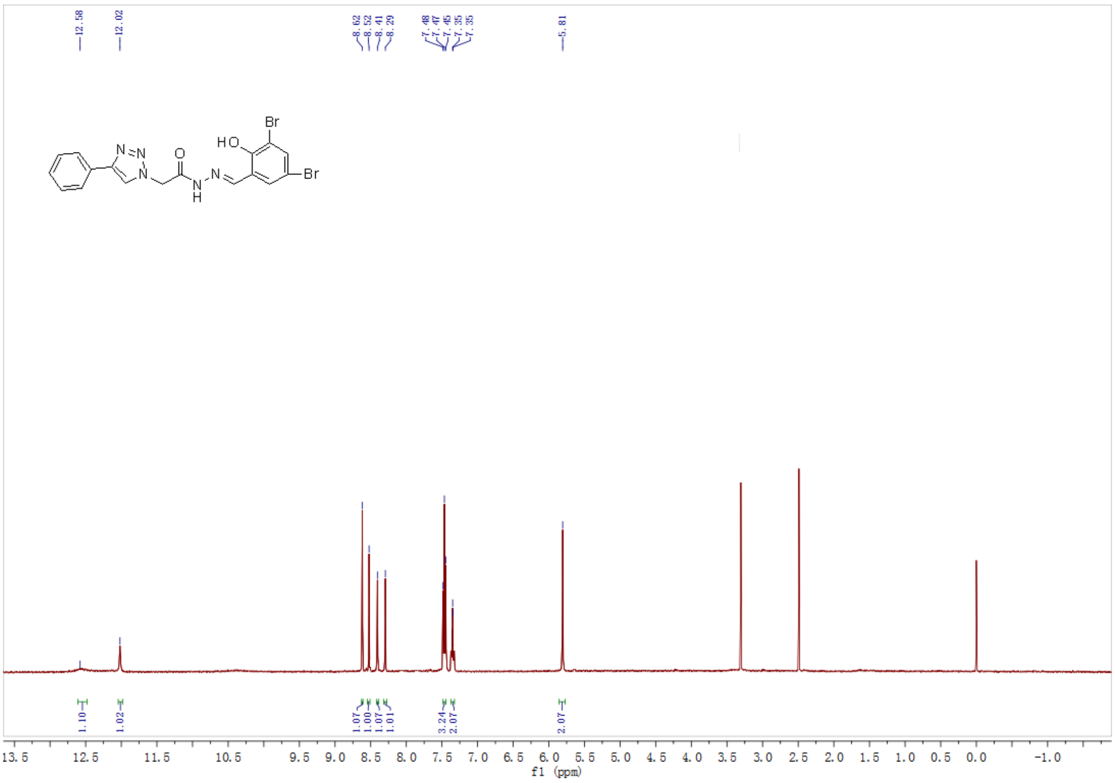

^{1}H-NMR spectrum of compound 11g

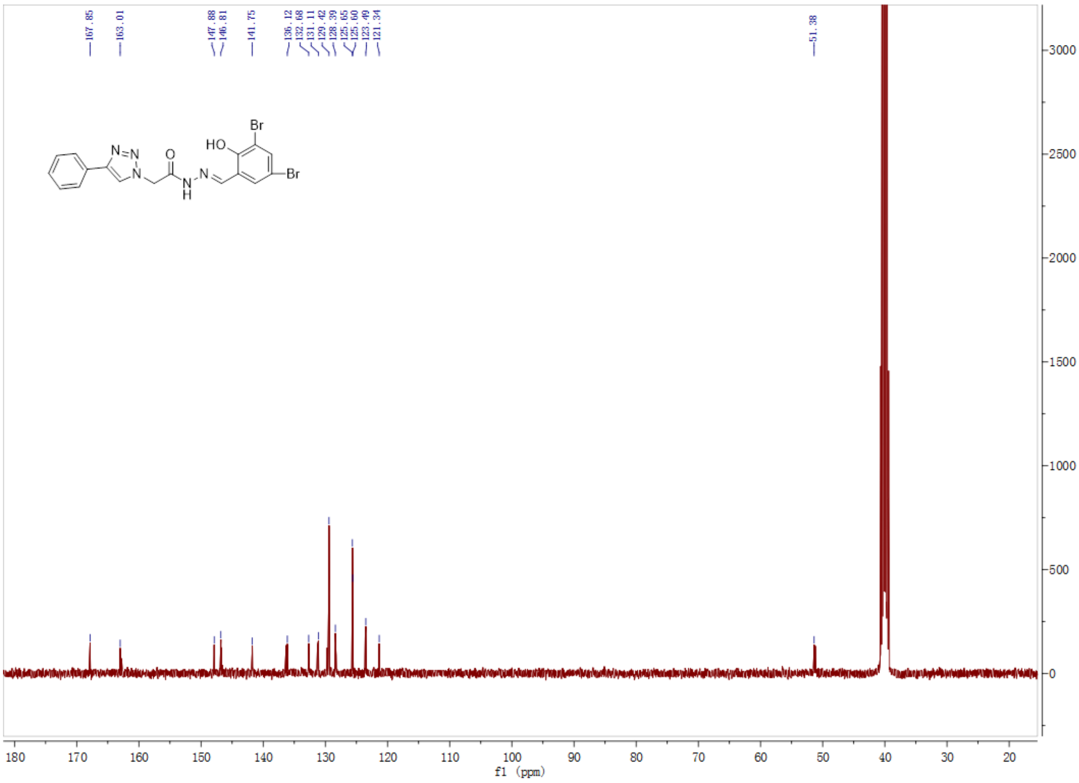

^{13}C-NMR spectrum of compound 11g

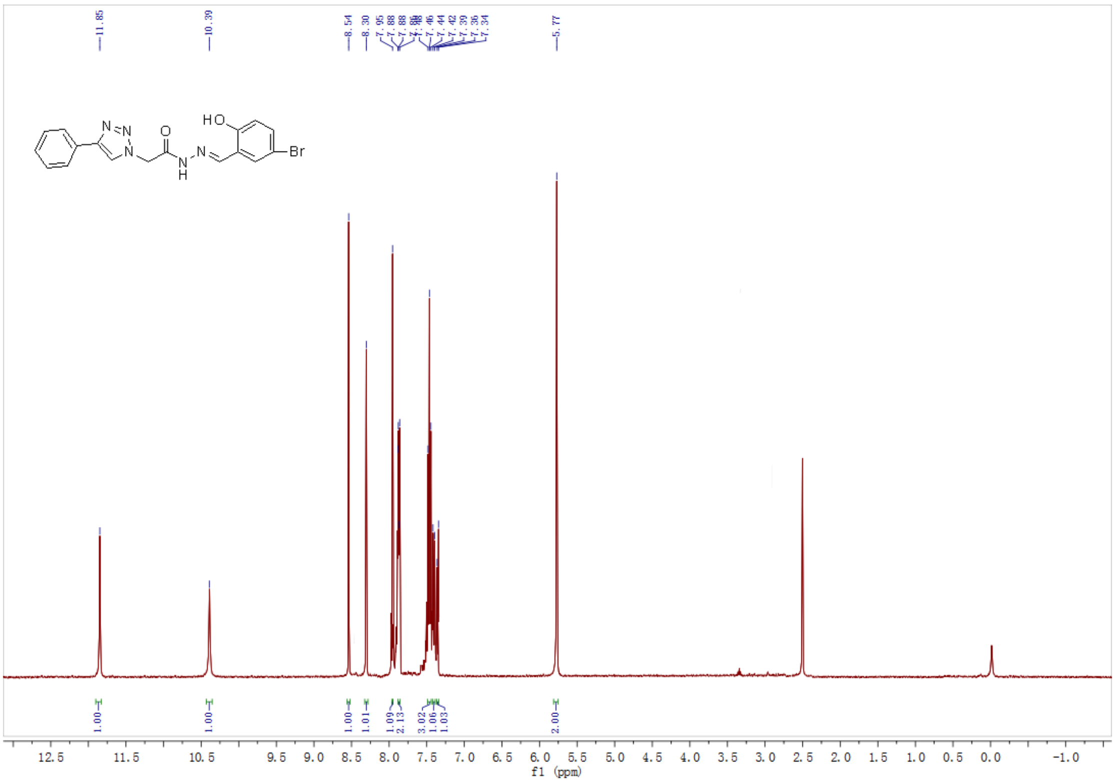

^{1}H-NMR spectrum of compound 11h

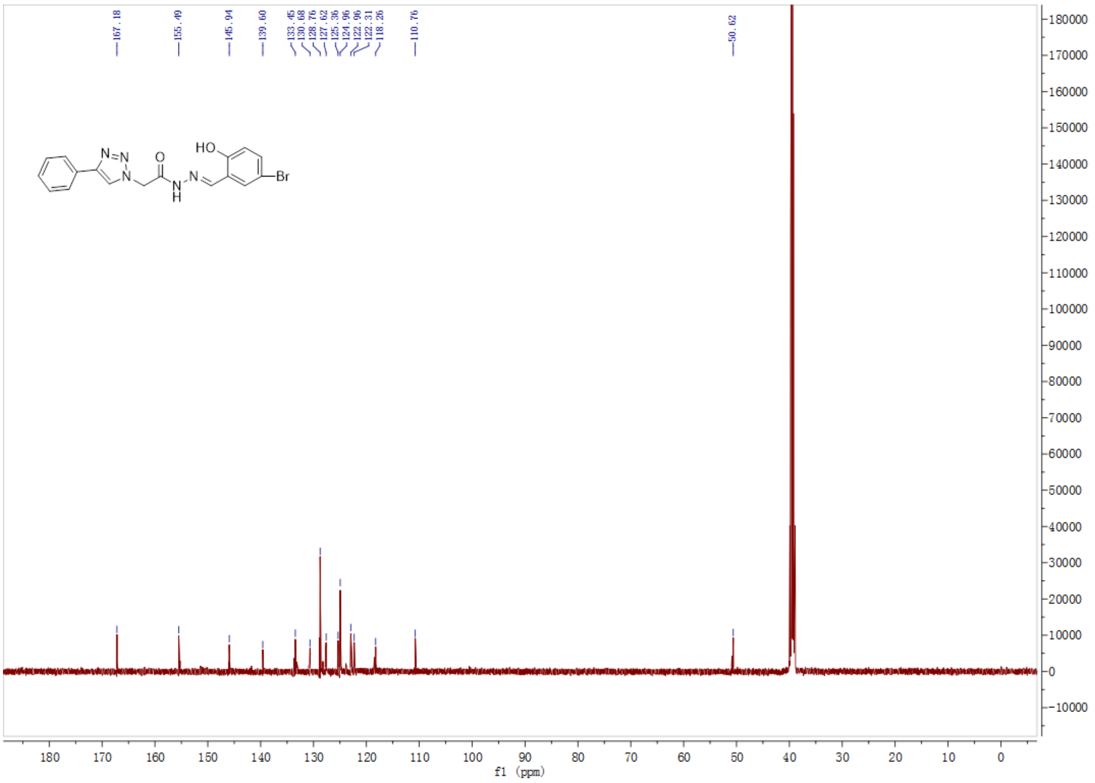

^{13}C-NMR spectrum of compound 11h

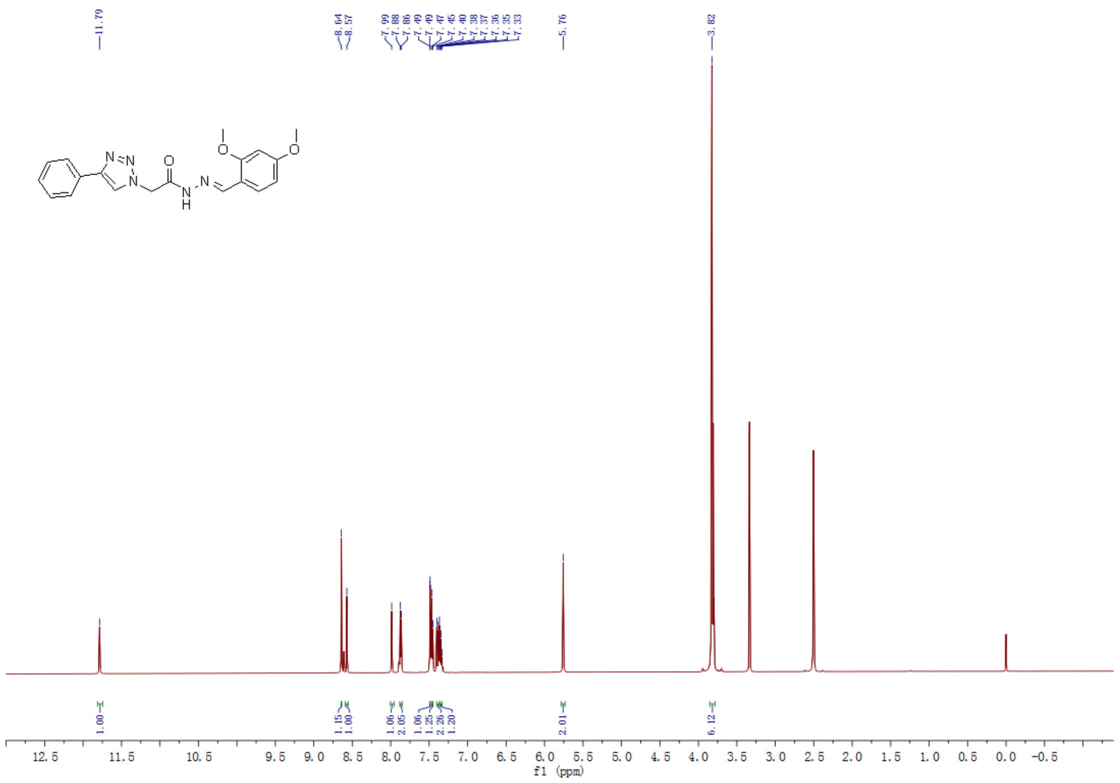

^{1}H-NMR spectrum of compound 11i

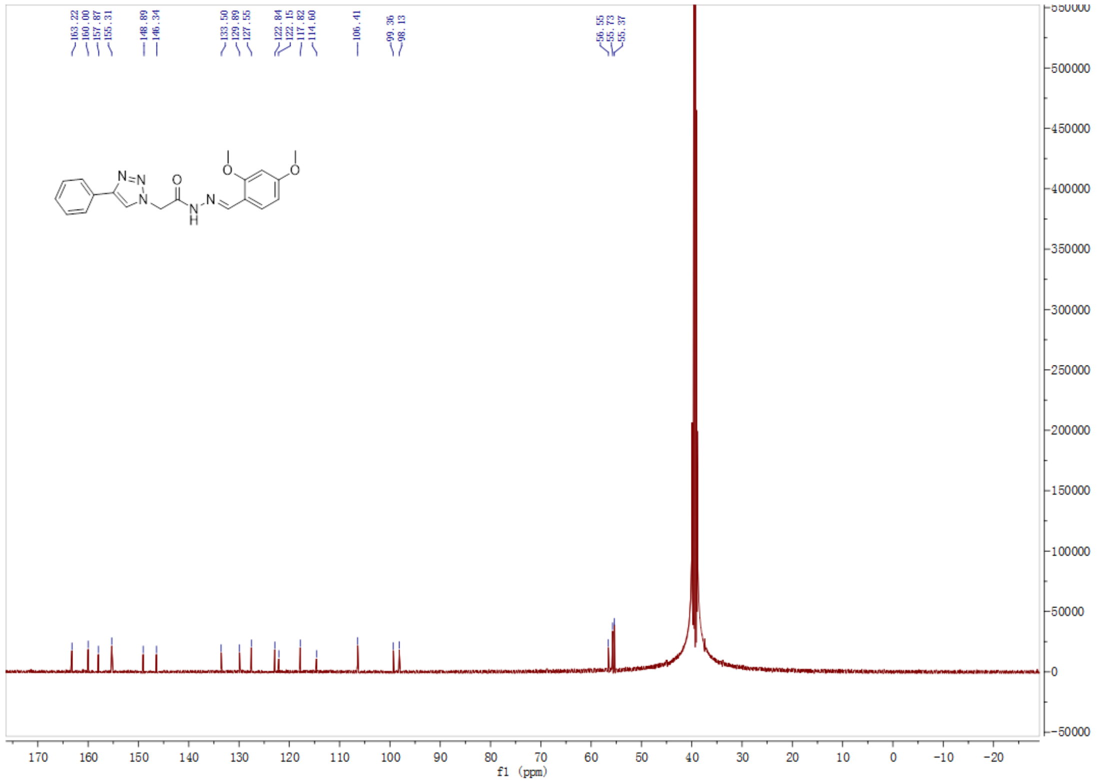

^{13}C-NMR spectrum of compound 11i

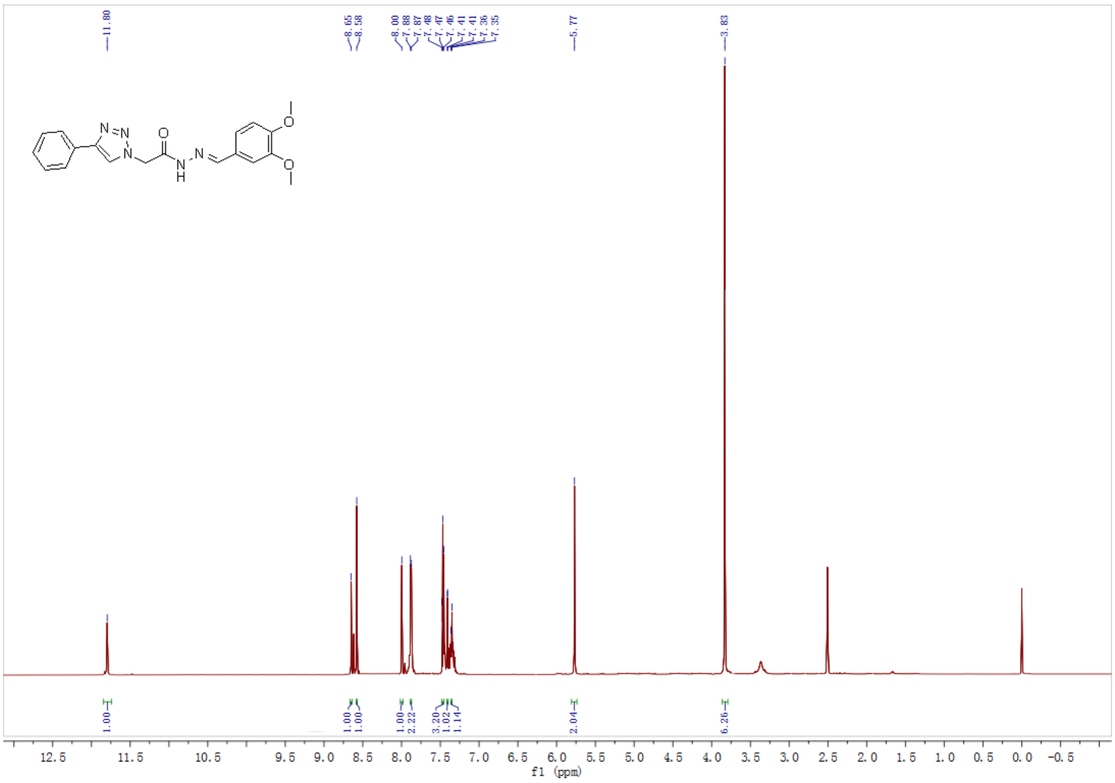

^{1}H-NMR spectrum of compound 11j

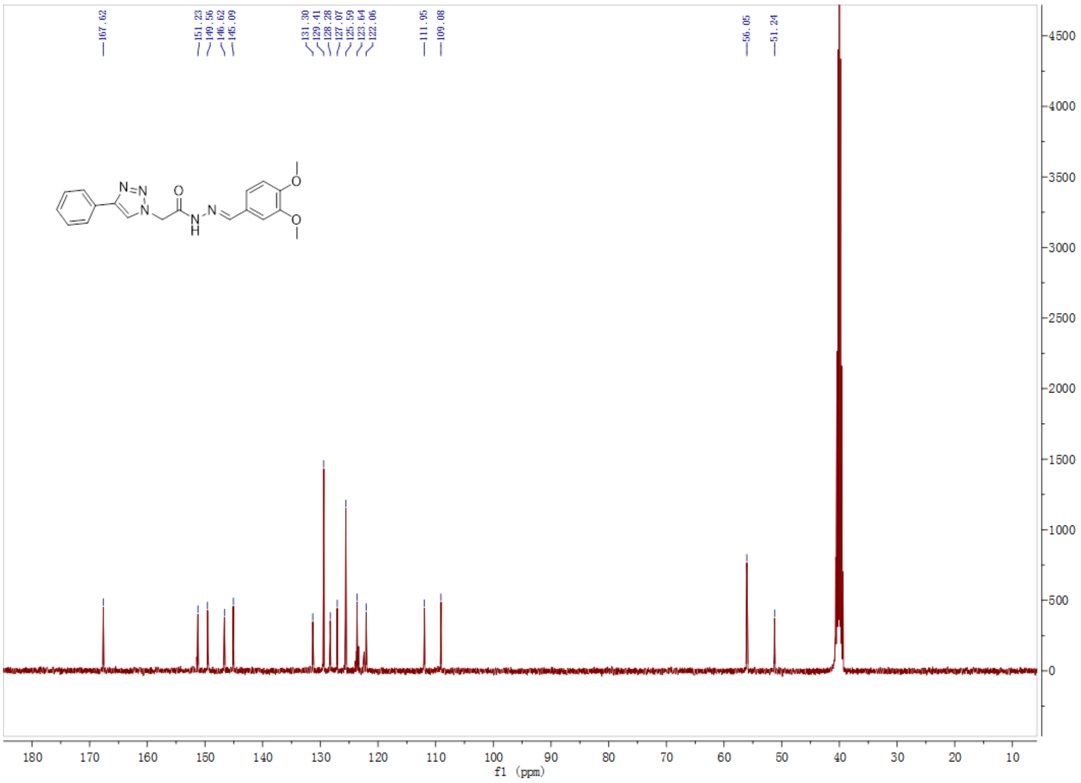

^{13}C-NMR spectrum of compound 11j

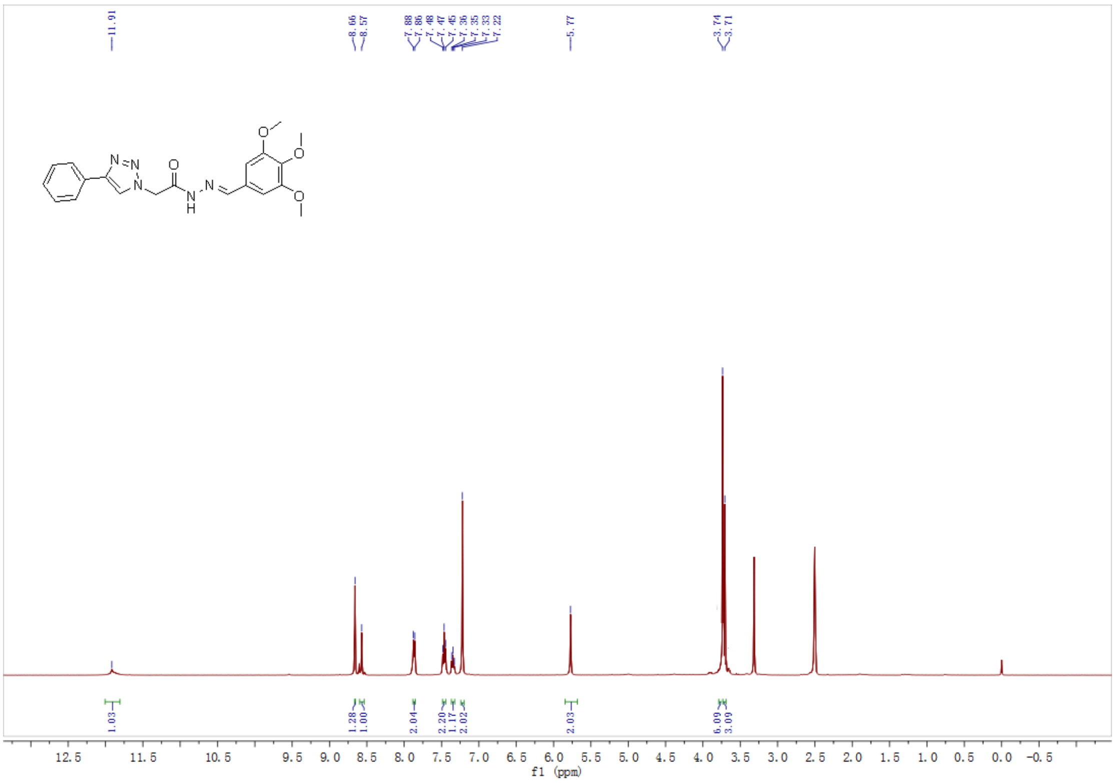

^{1}H-NMR spectrum of compound 11k

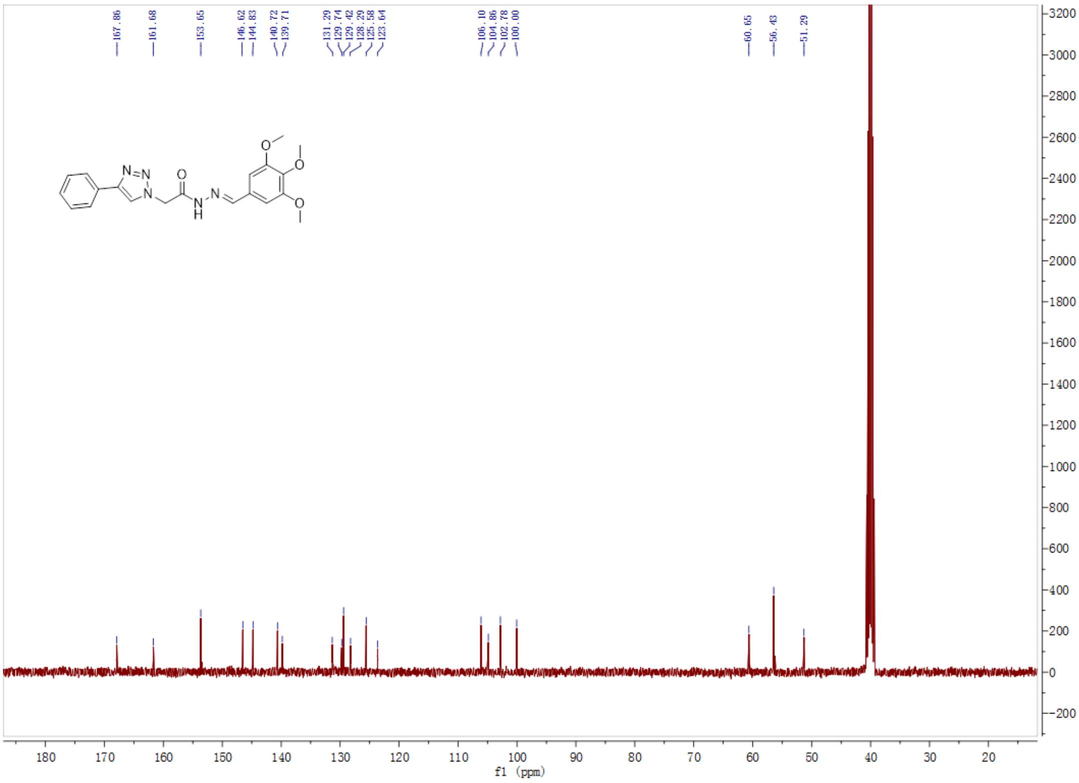

^{13}C-NMR spectrum of compound 11k
